# Supplementary material for: Cytoplasmic glycoengineering enables biosynthesis of nanoscale glycoprotein assemblies
Source: Nat Commun. 2019 Nov 27;10:5403. doi: 10.1038/s41467-019-13283-2 (PMC6881330; doi:10.1038/s41467-019-13283-2)
Supplement: Supplementary file 1 — Supplementary Information [file 41467_2019_13283_MOESM1_ESM.pdf]

SUPPLEMENTARY INFORMATION

CYTOPLASMIC GLYCOENGINEERING ENABLES BIOSYNTHESIS OF NANOSCALE GLYCOPROTEIN ASSEMBLIES

Hanne L.P. Tytgat[†1,4], Chia-wei Lin[†1,5], Mikail D. Levasseur[2], Markus B. Tomek[1], Christoph Rutschmann[1], Jacqueline Mock[1], Nora Liebscher[1], Naohiro Terasaka[2], Yusuke Azuma[2], Michael Wetter[1], Martin F. Bachmann[3], Donald Hilvert[2], Markus Aebi[1], Timothy G. Keys[1‡]

[1]Institute of Microbiology, ETH Zurich, 8093 Zurich, Switzerland

[2]Laboratory of Organic Chemistry, ETH Zurich, 8093 Zurich, Switzerland

[3]Department of Immunology, Inselspital, University of Bern, 3010 Bern, Switzerland

[4]Current address: Laboratory of Microbiology, Wageningen University, 6708 WE Wageningen, The Netherlands

[5]Current address: Functional Genomic Center Zurich, ETH Zurich, 8057 Zurich, Switzerland

[†]These authors contributed equally

[‡]To whom correspondence should be addressed: tim.keys@micro.biol.ethz.ch

SUPPLEMENTARY TABLES

Supplementary Table 1 - Glycosyltransferases used in this study.

GT	Activity	Source	Accession no	Reference
ApNGT	Glucosylation of N residues in an N-X-S/T sequon	*Actinobacillus pleuropneumoniae* strain AP76	ATN32182	Naegeli *et al.* 2014[1]
LgtB	Galactosylation of Glucose and GlcNAc residues	*Neisseria meningitidis* B MC58	ATN32181	Lau *et al.* 2010[2]
LgtA	GlcNAcylation of galactose	*N. meningitidis* B MC58	WP_050305168	Blixt *et al.* 1999[3]
FucTII	α1-2 fucosylation of lactose	*Helicobacter pylori*	AAD29868	Wang *et al.* 1999[4,5]
FutA	α1-3 fucosylation of lactosamine	*Helicobacter pylori*	WP_000487428	Ge *et al.* 1997
FKP	Bifunctional enzyme: L-fucokinase/GDP-fucose pyrophosphorylase	*Bacteroides fragilis*	WP_032533437	Baumgartner *et al.* 2013[6,7]
SynX-SiaB-SiaC	De novo biosynthesis of CMP-Neu5Ac	*N. meningitidis* MC58	AAF405375-7	This work
CstI	α2-3 sialylation of lactose	*Campylobacter jejuni* OH4384	WP_002855892	Chiu *et al*. 2007[8]
CstII	Dual function: addition of an α2-3 and an α2-8 linked sialic acid residue to a lactose primer	*C. jejuni* OH4382/84 with an 32 AA deletion and I53S mutation	ATN32180	Chiu *et al.* 2004[9]
NmB-polyST	Elongation of a disialylated primer with NmB capsule glycan	*N. meningitidis B* which was engineered	ATN32202	Keys *et al.* 2014[10]
NmC-polyST	Elongation of a disialylated primer with NmC capsule glycan	*N. meningitidis* C	WP_002247491	Peterson *et al.* 2011[11]

Supplementary Table 2 - Strains and plasmids

**Strain/Plasmid**	**Genotype**	**Reference or source**
**Strains**		
DH5α	F– Φ80*lac*ZΔM15 Δ(*lac*ZYA-*arg*F) U169 *rec*A1 *end*A1 *hsd*R17 (rK–, mK+) *pho*A *sup*E44 λ–*thi*–1 *gyr*A96 *rel*A1	Stratagene
W3110 *nan*AK *lac*Z	F-, λ^-, *IN(rrnD-rrnE)1*, *rph-1*, Δ*nan*A, Δ*nan*K, Δ*lac*Z	LimmaTech Biologics AG
**Plasmids - GTBbs**		
pMA1273	pSEVA331Bb, pSEVA331 with Biobrick multiple cloning site, pSEVA331, pBBR1 ori, Cm^R, negative control	AddGene, Florea *et al.* 2016[12]
pHT012	pSEVA341 with Biobrick multiple cloning site, pSEVA341, ColE1 ori, Cm^R, lacI insertion using KflI and SwaI sites, negative control	This work
pHT018	pSEVA331 with Biobrick multiple cloning site, pSEVA331, pBBR1 ori, Cm^R, BioBrick cargo: sfGFP	This work
pHT059	pHT018 with GTBb encoding ApNGT under control of lacUV5 promoter	This work
pMA993	pACYC-DUET with ApNGT under control of a lacUV5 promoter, Cm^R	Keys *et al.* 2017[13]
pHT060	pHT018 with GTBbs encoding ApNGT and LgtB under control of lacUV5 promoters	This work
pHT091	pHT018 with GTBbs encoding ApNGT, LgtB, CstI and SynX-SiaB-SiaC operon under control of lacUV5 promoters	This work
pHT081	pHT018 with GTBbs encoding ApNGT, LgtB, CstI and SynB under control of lacUV5 promoters	This work
pHT064	pHT018 with GTBbs encoding ApNGT, LgtB, FucTII and FKP under control of lacUV5 promoters	This work
pHT089	pHT018 with GTBbs encoding ApNGT, LgtB and SynX-SiaB-SiaC operon under control of lacUV5 promoters, and CstII under control of a T5 promoter	This work
pHT072	pHT018 with GTBbs encoding ApNGT, LgtB, SynX-SiaB-SiaC operon and NmCPST under control of lacUV5 promoters, and CstII under control of a T5 promoter	This work
pHT075	pHT018 with GTBbs encoding ApNGT, LgtB, SynX-SiaB-SiaC operon and NmBPST under control of lacUV5 promoters, and CstII under control of a T5 promoter	This work
pHT080	pHT018 with GTBbs encoding ApNGT and LgtB, under control of lacUV5 promoters, and LgtA under control of a T5 promoter	This work
pHT093	pHT018 with GTBbs encoding ApNGT, LgtB, FutA, FKP under control of lacUV5 promoters, and LgtA under control of a T5 promoter	This work

SUPPLEMENTARY FIGURES

a

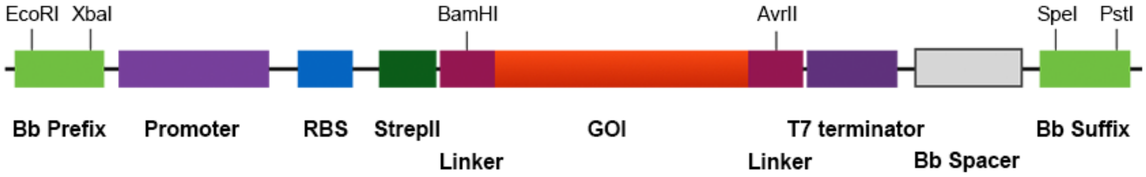

b

GAATTCGCGGCCGCTTCTAGAGCTAGGCACCCCAGGCTTT
ACACTTTATGCTTCCGGCTCGTATAATGTGTGGAATTGTGA
GCGGATAACAATTCCCCTCGAGAAATAATTTTGTTTAACTT
TAATAAGGAGATATACCATGGATAGCTGGAGCCACCCGCA
GTTCGAAAAAGGCGCCCTGGATATCGGATCCNNNNNNNN
NNNNNTTAACCTAGGCTGCTGCCACCGCTGAGCAATAACT
AGCATAACCCCTTGGGGCCTCTAAACGGGTCTTGAGGGGT
TTTTTGAGGTTCTGTTAAGTAACTGAACCCAATGTCGTTAG
TGACGCTTACCTCTTAAGAGGTCACTGACCTAACATACTAG
TAGCGGCCGCTGCAG

Bb prefix (EcoRI and XbaI)
Promoter region
RBS
Start translation
StrepII-tag
Linker flanking GOI
Gene of interest
T7 terminator
Bb spacer
Bb suffix (SpeI and PstI)

Supplementary Figure 1 – BioBrick expression cassette. (**A**) The expression cassette is flanked by the biobrick (Bb) pre- and suffix, harboring isocaudameric restriction sites. The cassette consists of an inducible promoter (T5, Ptac, Plac or PlacUV5), a ribosome binding site (RBS), a Strep-tagII that may be genetically fused to the gene of interest (GOI), and a T7 terminator. (**B**) Nucleotide sequence of the expression cassette.

Glycotag sfGFP, single site
MGSHHHHHHHHHHGSSKGEELFTGVVPILVELDGDVNGHKFSVRGEGEGDATNGKLTLKFICTTGKLPVPWPTLVTTLTYGVQ
CFSRYPDHMKRHDFFKSAMPEGYVQERTISFKDDGTYKTRAEVKFEGDTLVNRIELKGIDFKEDGNILGHKLEYNFNSHNVYITA
DKQKNGIKANFKIRHNVEDGSVQLADHYQQNTPIGDGPVLLPDNHYLSTQSVLSKDPNEKRDHMVLLEFVTAAGITKTSAHAT
ASGAHATAGSA**NAT**AWSHPQFEK

Glycotag sfGFP, [GNAT]$_5$
MGSHHHHHHHHHHGSSKGEELFTGVVPILVELDGDVNGHKFSVRGEGEGDATNGKLTLKFICTTGKLPVPWPTLVTTLTYGVQ
CFSRYPDHMKRHDFFKSAMPEGYVQERTISFKDDGTYKTRAEVKFEGDTLVNRIELKGIDFKEDGNILGHKLEYNFNSHNVYITA
DKQKNGIKANFKIRHNVEDGSVQLADHYQQNTPIGDGPVLLPDNHYLSTQSVLSKDPNEKRDHMVLLEFVTAAGITKTSRG**NA**
TG**NAT**G**NAT**G**NAT**G**NAT**WSHPQFEK

Glycotag sfGFP, [GANATA]$_5$
MGSHHHHHHHHHHGSSKGEELFTGVVPILVELDGDVNGHKFSVRGEGEGDATNGKLTLKFICTTGKLPVPWPTLVTTLTYGVQ
CFSRYPDHMKRHDFFKSAMPEGYVQERTISFKDDGTYKTRAEVKFEGDTLVNRIELKGIDFKEDGNILGHKLEYNFNSHNVYITA
DKQKNGIKANFKIRHNVEDGSVQLADHYQQNTPIGDGPVLLPDNHYLSTQSVLSKDPNEKRDHMVLLEFVTAAGITKTSRGA**N**
ATAGA**NAT**AGA**NAT**AGA**NAT**AGA**NAT**AWSHPQFEK

Glycotag sfGFP, [TAGANATA]$_5$
MGSHHHHHHHHHHGSSKGEELFTGVVPILVELDGDVNGHKFSVRGEGEGDATNGKLTLKFICTTGKLPVPWPTLVTTLTYGVQ
CFSRYPDHMKRHDFFKSAMPEGYVQERTISFKDDGTYKTRAEVKFEGDTLVNRIELKGIDFKEDGNILGHKLEYNFNSHNVYITA
DKQKNGIKANFKIRHNVEDGSVQLADHYQQNTPIGDGPVLLPDNHYLSTQSVLSKDPNEKRDHMVLLEFVTAAGITKTSRTAG
A**NAT**ATAGA**NAT**ATAGA**NAT**ATAGA**NAT**ATAGA**NAT**AWSHPQFEK

Glycotag AP205cp
MANKPMQPITSTANKIVWSDPTRLSTTFSASLLRQRVKVGIAELNNVSGQYVSVYKRPAPKPEGCADACVIMPNENQSIRTVIS
GSAENLATLKAEWETHKRNVDTLFASGNAGLGFLDPTAAIVSSDTTAGSGGAHATA**NAT**AHATWSHPQFEK

AaLS-13
MEIYEGKLTAEGLRFGIVASRFNHALVGRLVEGAIDCIVRHGGREEDITLVCVPGSWEIPVAAGELARKEDIDAVIAIGVLIEGAEP
HFDYIASEVSKGLANLSLELRKPISFGDITDDELEEAIECAGTEHGNKGWEAALSAIEMANLFKSLRLEHHHHHH

AaLS-13-GI
MEIYEGKLTAEGLRFGIVASRFNHALVGRLVEGAIDCIVRHGGREEDITLVCVPGSWEIPVAAGELARKEDIDAVIAIGVLIEGAEP
HFDYIASEVSKGLANLSLELRKPISFGDITDDELEEAIECAGTEHGNKGWEAALSAIEMANLFKSLRLEGSGSGAHATA**NAT**AHAS
HHHHHH

AaLS-13-GIII
MEIYEGKLTAEGLRFGIVASRFNHALVGRLVEGAIDCIVRHGGREEDITLVCVPGSWEIPVAAGELARKEDIDAVIAIGVLIEGAEP
HFDYIASEVSKGLANLSLELRKPISFGDITDDELEEAIECAGTEHGNKGWEAALSAIEMANLFKSLRLEGSGSGA**NAT**A**NAT**A**NAS**
HHHHHH

Glycotag I53-50-v4
Trimer subunit:
MTMEELFKRHTIVAVLRANSVEEAIEKAVAVFAGGVHLIEITFTVPDADTVIKALSVLKEDGAIIGAGTVTSVDQCRKAVESGAEFI
VSPHLDEEISQFCKEKGVFYMPGVMTPTELVKAMKLGHDILKLFPGEVVGPQFVKAMKGPFPNVKFVPTGGVNLDNVCKWFK
AGVLAVGVGNALVKGNPDKVREKAKKFVKKIRGCTEGSWSHPQFEK
Pentamer subunit:
MGSS**NAT**GSNQHSQKDQETVRIAVVRARWHAFIVDACVSAFEAAMRKIGGERFAVDVFDVPGAYEIPLHARTLAKTGRYGAV
LGTAFVVNGGIYRHEFVASAVIDGMMNVQLDTGVPVLSAVLTPHNYDKSNAKTLLFLALFAVKGMEAARACVEILAAREKIAAG
SFEHHHHHH

Supplementary Figure 2 - Amino acid sequences of protein substrates used in this study. Features are marked as follows: poly-histidine tag; strep-tag II; linker sequence; **glycosylation sequon**.

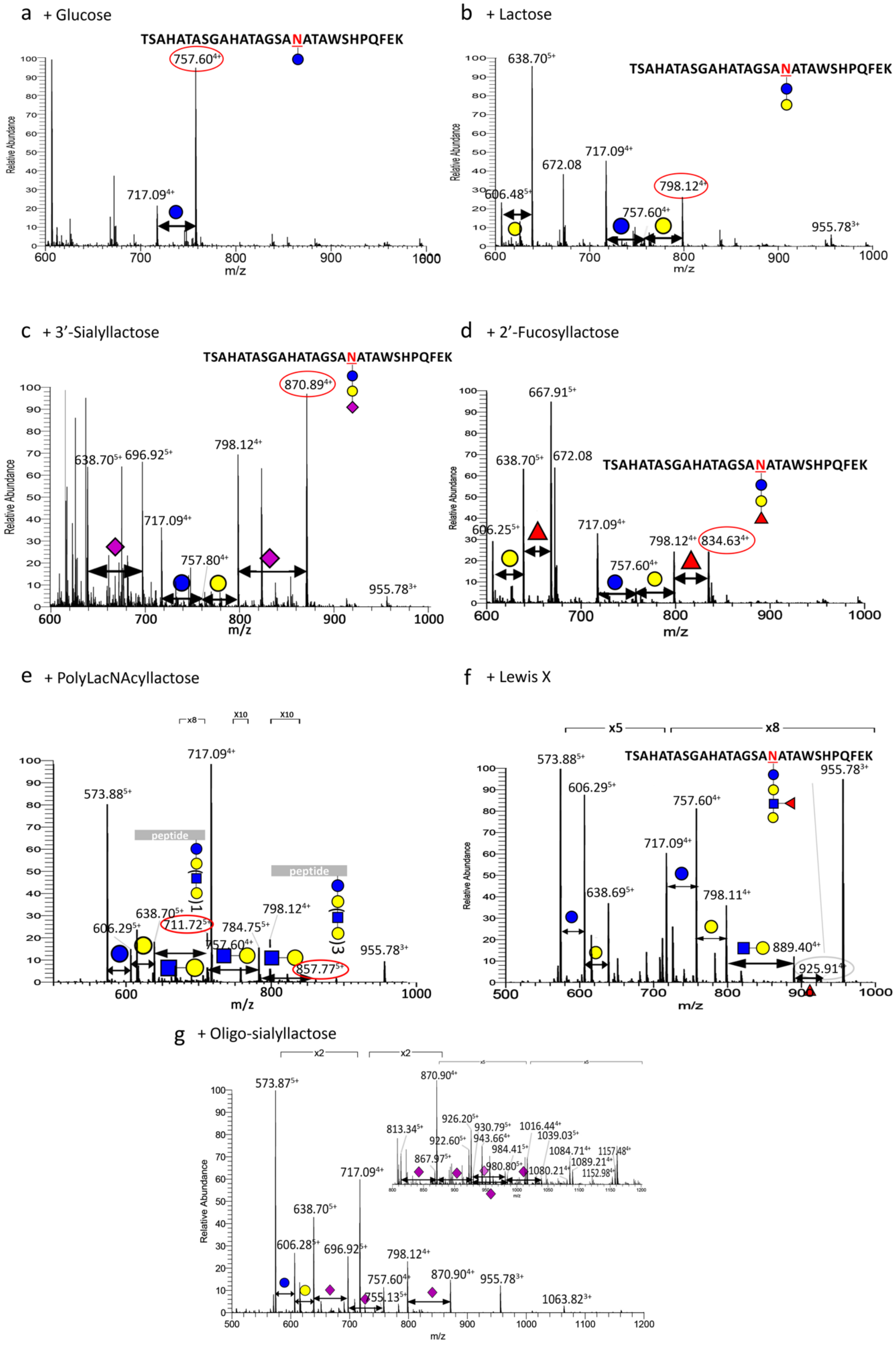
a + Glucose
TSAHATASGAHATAGSANATAWSHPQFEK
757.60⁴⁺
717.09⁴⁺
Relative Abundance
m/z
b + Lactose
TSAHATASGAHATAGSANATAWSHPQFEK
638.70⁵⁺
606.48⁵⁺
672.08
717.09⁴⁺
757.60⁴⁺
798.12⁴⁺
955.78³⁺
c + 3′-Sialyllactose
TSAHATASGAHATAGSANATAWSHPQFEK
870.89⁴⁺
638.70⁵⁺
696.92⁵⁺
798.12⁴⁺
717.09⁴⁺
757.80⁴⁺
955.78³⁺
d + 2′-Fucosyllactose
TSAHATASGAHATAGSANATAWSHPQFEK
667.91⁵⁺
638.70⁵⁺
672.08
606.25⁵⁺
717.09⁴⁺
798.12⁴⁺
834.63⁴⁺
757.60⁴⁺
e + PolyLacNAcyllactose
x8
X10
X10
717.09⁴⁺
573.88⁵⁺
peptide
638.70⁵⁺
606.29⁵⁺
711.72⁵⁺
798.12⁴⁺
784.75⁵⁺
757.60⁴⁺
955.78³⁺
857.77⁵⁺
f + Lewis X
x5
x8
573.88⁵⁺
TSAHATASGAHATAGSANATAWSHPQFEK
955.78³⁺
606.29⁵⁺
757.60⁴⁺
717.09⁴⁺
638.69⁵⁺
798.11⁴⁺
889.40⁴⁺
925.91⁴⁺
g + Oligo-sialyllactose
x2
x2
x5
573.87⁵⁺
870.90⁴⁺
926.20⁵⁺
813.34⁵⁺
930.79⁵⁺
1016.44⁴⁺
922.60⁵⁺
943.66⁴⁺
1039.03⁵⁺
984.41⁵⁺
867.97⁵⁺
1084.71⁴⁺
1157.48⁴⁺
980.80⁵⁺
1089.21⁴⁺
1080.21⁴⁺
1152.98⁴⁺
717.09⁴⁺
638.70⁵⁺
606.28⁵⁺
696.92⁵⁺
798.12⁴⁺
757.60⁴⁺
870.90⁴⁺
955.78³⁺
755.13⁵⁺
1063.82³⁺

Supplementary Figure 3 – Cytoplasmic protein glycosylation. GFP carrying a single glycosylation site was co-expressed with a prototype glycosylation pathway. The (glyco)protein product was affinity purified, tryptic peptides were prepared and analyzed by nanoLC-MS/MS. Mass spectra show the glycosylation profile of proteins modified with: (**a**) glucose, (**b**) lactose, (**c**) 3'-sialyllactose, (**d**) 2'-fucosyllactose, (**e**) N-acetyllactosamine (LacNAc) repeats, (**f**) Lewis x, (**g**) oligo-sialyllactose. MS spectra are the sum of all modified and unmodified peptides (with 3+, 4+ or 5+ charge states) of sequence: TSAHATASGAHATAGSANATAWSHPQFEK (glycosylation site underlined). Sugar assignments are based on the observed mass and expected products of the respective glycosylation pathway. Red circled m/z species correspond to the target glycan product. The structure of these species was supported by MS/MS analysis (as shown in Supplementary Figure 4).

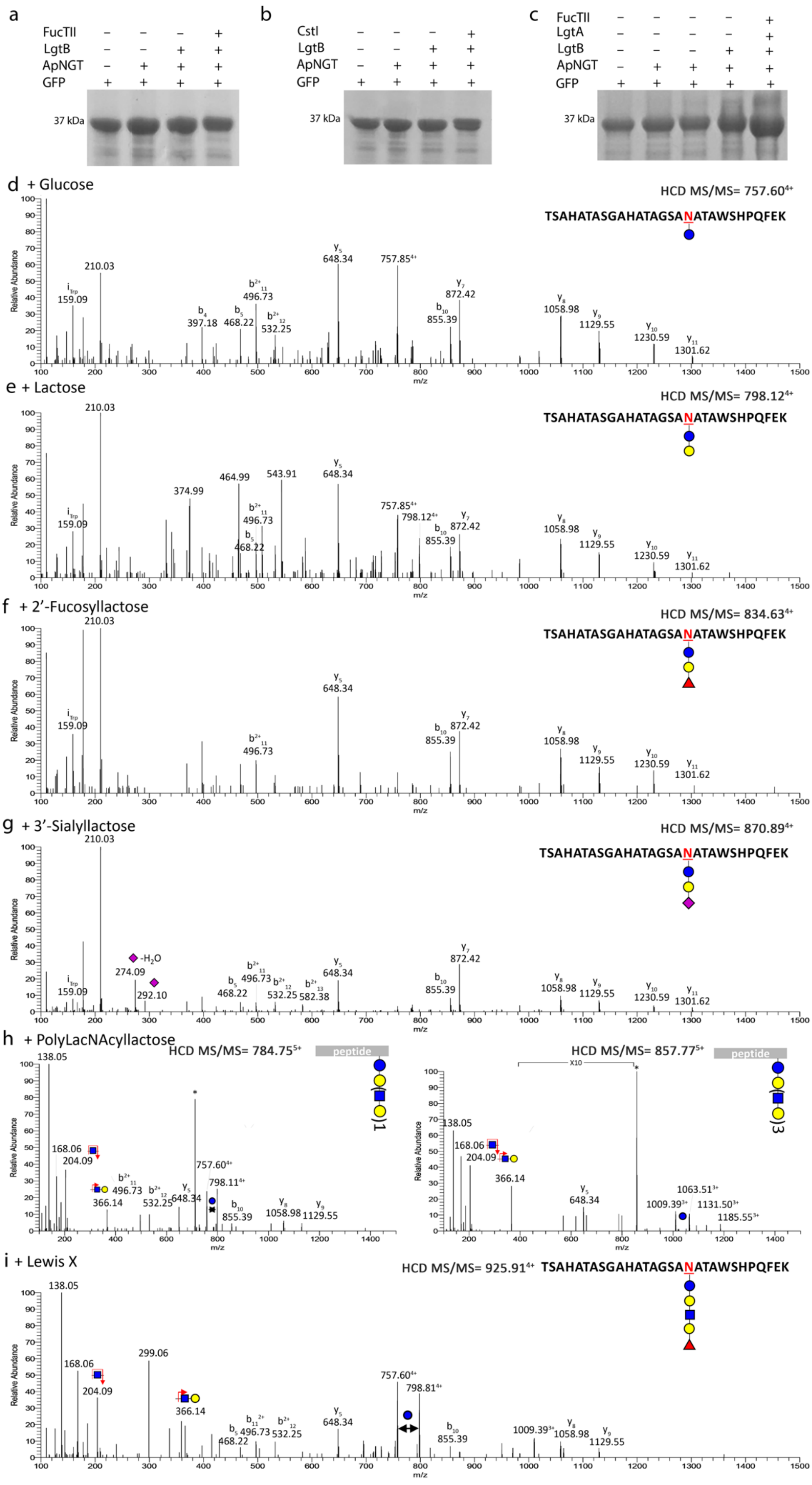
a
FucTII
LgtB
ApNGT
GFP
37 kDa
b
CstI
LgtB
ApNGT
GFP
37 kDa
c
FucTII
LgtA
LgtB
ApNGT
GFP
37 kDa
d + Glucose
HCD MS/MS= 757.60⁴⁺
TSAHATASGAHATAGSANATAWSHPQFEK
e + Lactose
HCD MS/MS= 798.12⁴⁺
TSAHATASGAHATAGSANATAWSHPQFEK
f + 2'-Fucosyllactose
HCD MS/MS= 834.63⁴⁺
TSAHATASGAHATAGSANATAWSHPQFEK
g + 3'-Sialyllactose
HCD MS/MS= 870.89⁴⁺
TSAHATASGAHATAGSANATAWSHPQFEK
h + PolyLacNAcyllactose
HCD MS/MS= 784.75⁵⁺
peptide
HCD MS/MS= 857.77⁵⁺
peptide
i + Lewis X
HCD MS/MS= 925.91⁴⁺
TSAHATASGAHATAGSANATAWSHPQFEK
Relative Abundance
m/z

Supplementary Figure 4 – Analysis of cytoplasmically produced glycoproteins. Coomassie stained denaturing PAGE (**a-c**) and HCD MS/MS spectra (**d-i**) of glycoproteins presented in **Figure 1** and **Supplementary Figure 3**. The HCD MS/MS spectra of (**d**) glucose-; (**e**) lactose-; (**f**) 2'fucosyllactose; (**g**) 3'sialyllactose; (**h**) LacNAc repeats; and (**i**) Lewis X modified peptides from GFP with C-terminal glycotag.

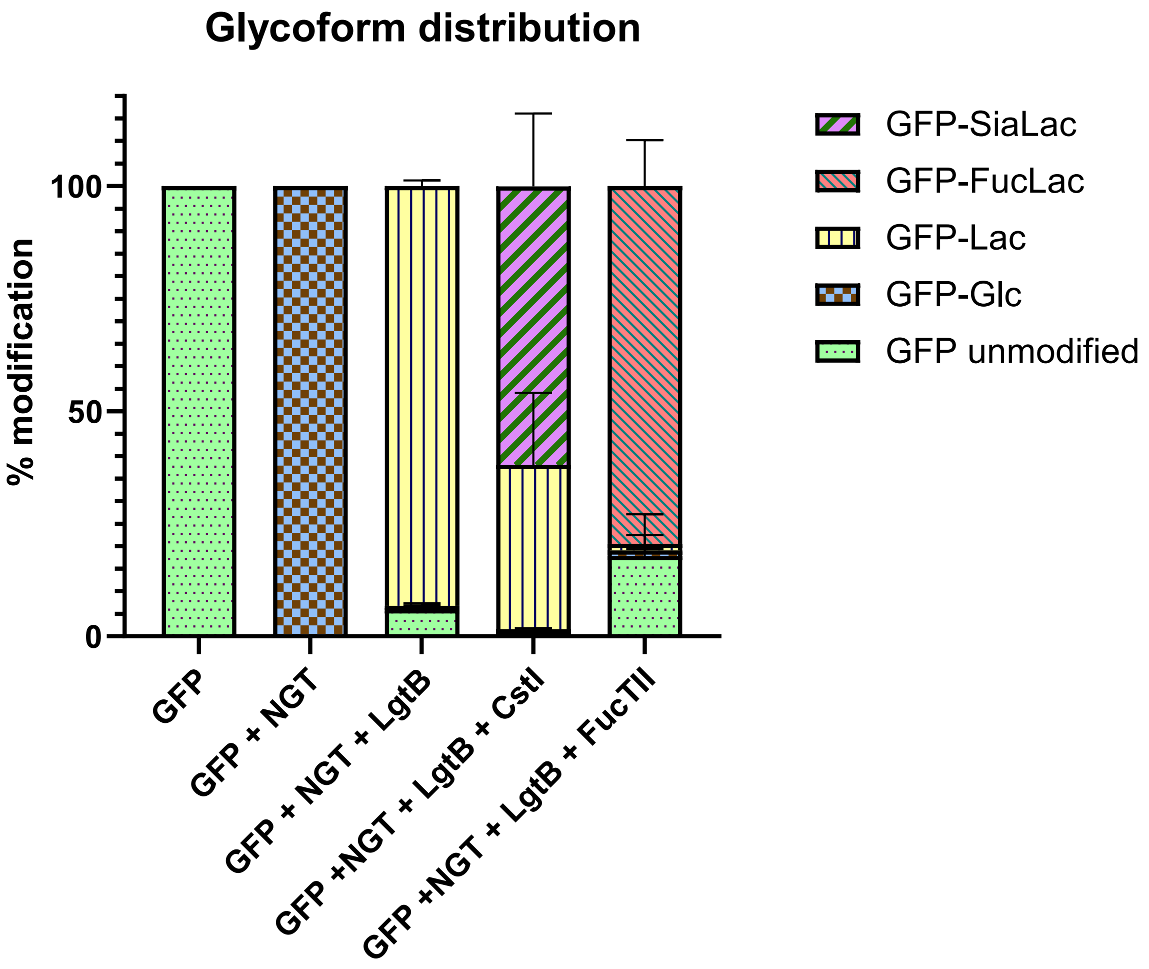

Supplementary Figure 5 - Distribution of glycoforms on sfGFP. The target protein was co-expressed with pathways for modification of asparagine residues with glucose (Glc), lactose (Lac), 3'-sialyllactose (SiaLac), or 2'-fucosyllactose (FucLac). Total glycoprotein products from three biological replicates were affinity purified and analyzed by intact protein MS. The graph depicts the average (mean, error bars are SD, n=3) percentage of total protein modified with no sugar ("unmodified"), Glc, Lac, SiaLac, or FucLac. Quantification of each species is based on peak heights in the intact protein mass spectra. Full spectra are provided in the **Source data file** associated with this manuscript.

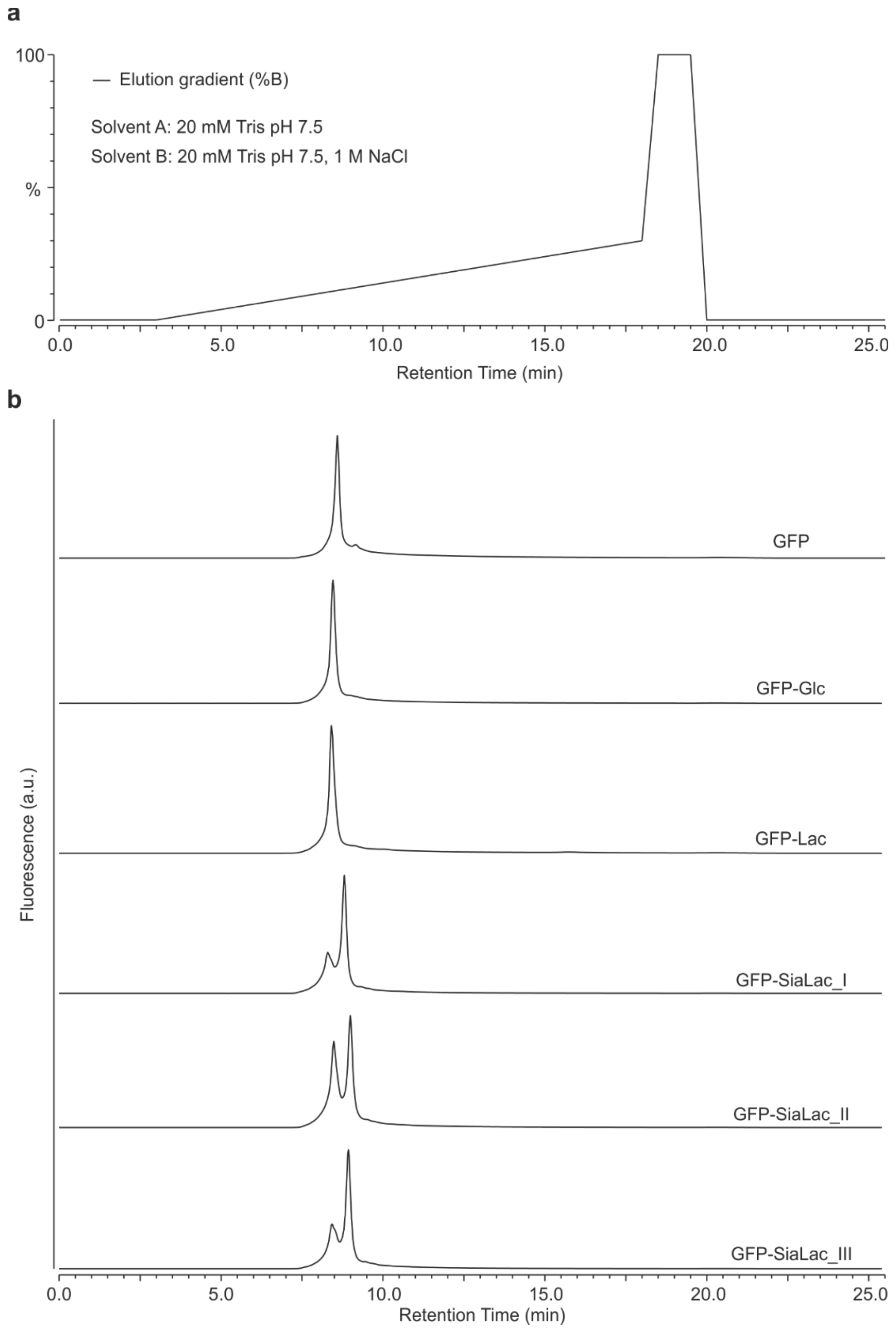

Supplementary Figure 6 – Analysis of GFP modified with the 3'-sialyllactose (SiaLac) pathway. Purified glycoproteins were separated on a ProPac SAX-10 column at a flow rate of 1 ml/min in a gradient of NaCl at pH 7.5. Elution of GFP was monitored with an online fluorescence detector (Ex. 385, Em. 410). (**a**) The solvent gradient used for chromatography. (**b**) GFP samples including unmodified protein, as well as GFP modified with neutral sugars (Glc and Lac), and three biological replicates of products of the SiaLac pathway. GFP carrying the complete SiaLac structure is retained on the column for approximately 30 seconds longer than protein that is unmodified or modified with neutral sugars.

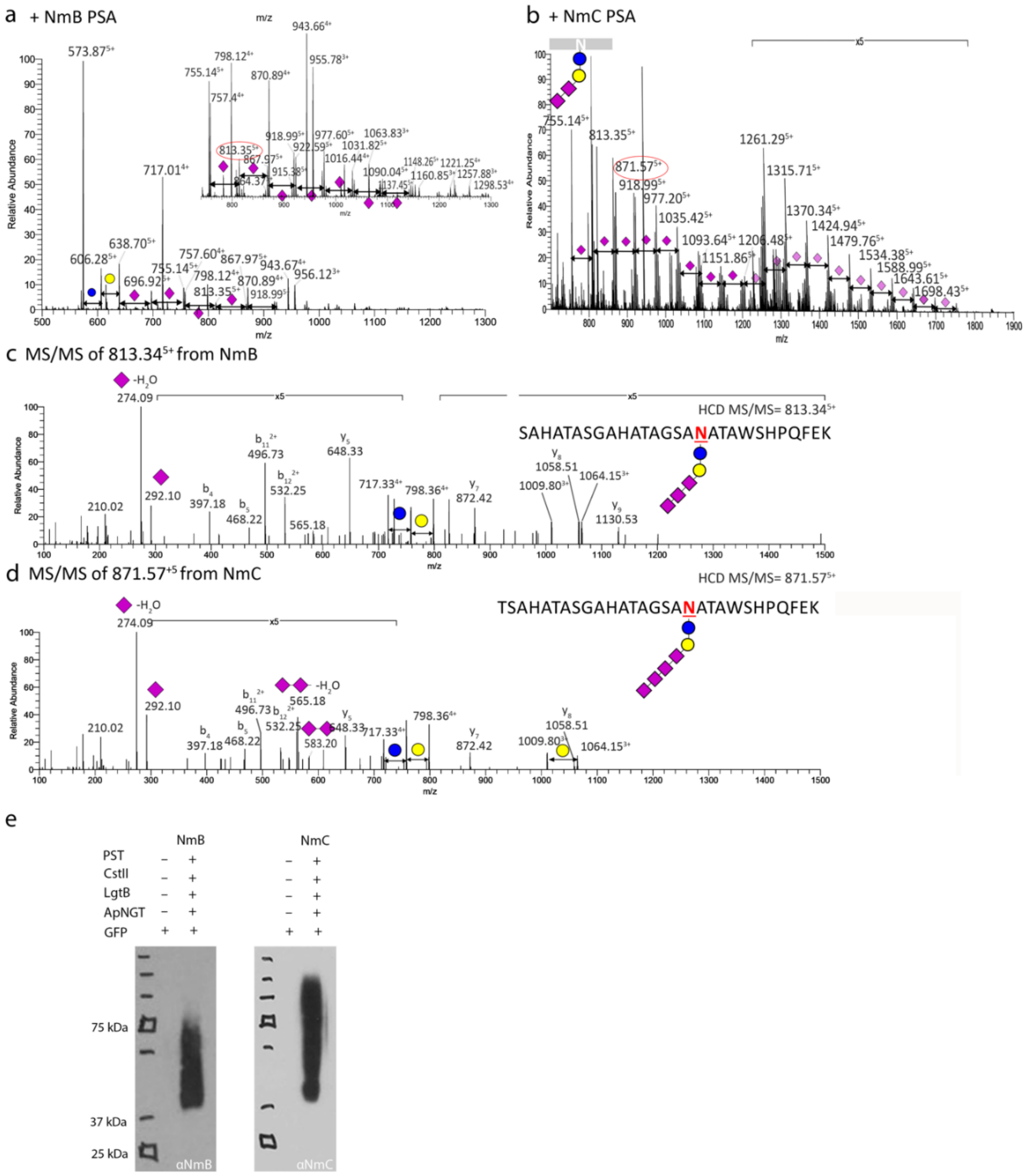

Supplementary Figure 7 – Structural characterization of CPS_{NmB} and CPS_{NmC} modified GFP. GFP carrying a single glycosylation site (TSAHATASGAHATAGSANATAWSHPQFEK) was co-expressed with a PSA glycosylation pathway. The (glyco)protein product was affinity purified, tryptic peptides were prepared and analysed by nanoLC-MS/MS. Mass spectra show the glycosylation profile of proteins modified with (**a**) CPS_{NmB} and (**b**) CPS_{NmC}. MS spectra are the sum of all modified and unmodified peptides (with 3+, 4+ or 5+ charge states). Sugar assignments are based on the observed mass and expected products of the respective glycosylation pathway. Red circles indicate m/z species corresponding to the target glycan product. The structure was further supported via annotated HCD MS/MS spectra of *m/z* species from **a** & **b,** shown in (**c** & **d**) . The *m/z* species analyzed are those annotated as tri- and tetra-sialyllactose modified peptides from GFP co-expressed with the (**c**) NmB- and (**d**) NmC-CPS pathways respectively. The diagnostic ions of sialic acid, $[NeuAc]^+$ 292.10 and $[NeuAc\text{-}H2O]^+$ 274.09, and disialic acid, $[2NeuAc]^+$ 583.2 and $[NeuAc\text{-}H2O]^+$ 565.18, were observed in all precursor ions with the mass corresponding to oligo- or polysialic acid structure. (**e**) Regiospecificity of the glycosidic linkages within the polysaccharide was confirmed by immunoblotting with capsule specific anti-sera.

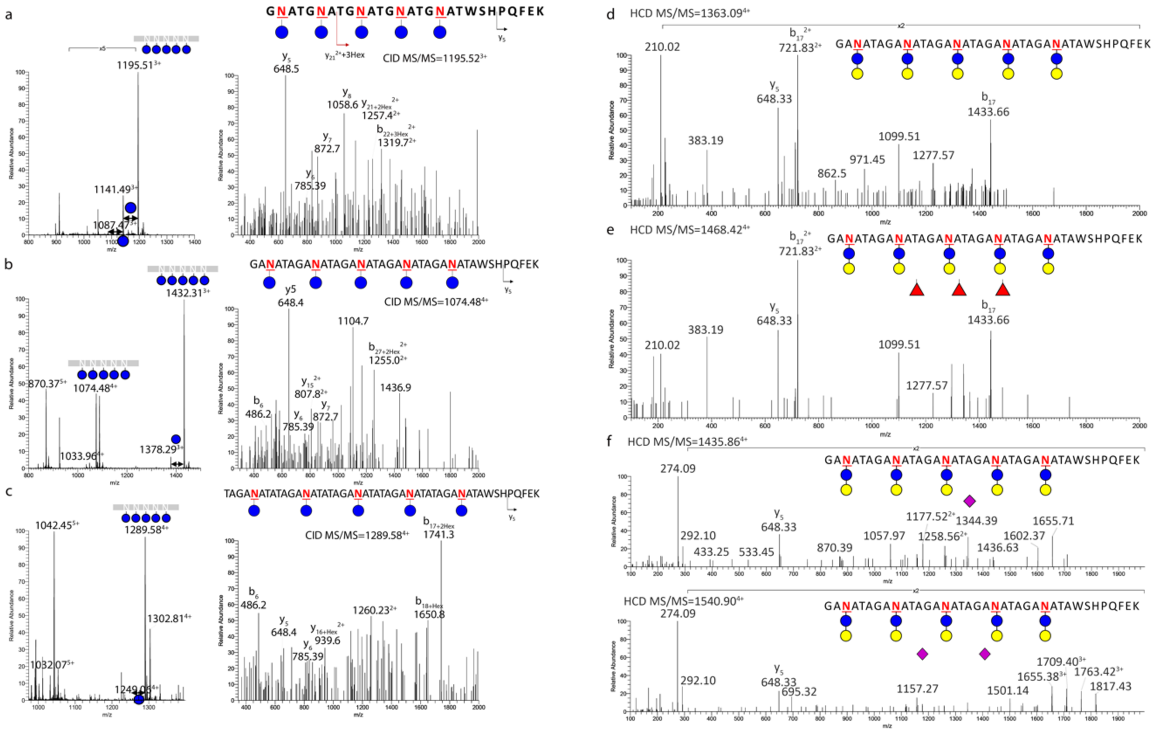

Supplementary Figure 8 – MS analysis of multivalent glycopolymers produced in the cytoplasm. Substrate proteins bearing pentavalent glycotags with sequence (**a**) [GNAT]$_5$, (**b**) [GANATA]$_5$, and (**c**) [TAGANATA]$_5$ were co-expressed with the NGT. The MS profile of tryptic peptides with the repeat sequence are shown in the left panel (**a**, **b**, and **c**). Corresponding CID-MS/MS spectra are in the right panel (**a**, **b**, and **c**). The HCD-MS/MS spectra of *m/z* species from Figure 2c-e; pentavalent glycopeptides with sequence [GANATA]$_5$ and modified with (**d**) lactose, (**e**) 2'fucosyllactose, and (**f**) 3'sialyllactose. Hovering sugars indicate the precise site of attachment was not able to be determined from the HCD spectra. Exceptionally, the data presented in (**a**)-(**c**) were collected on an LTQ-Orbitrap Velos mass spectrometer. All other data MS experiments were performed on a QExactive Orbitrap mass spectrometer.

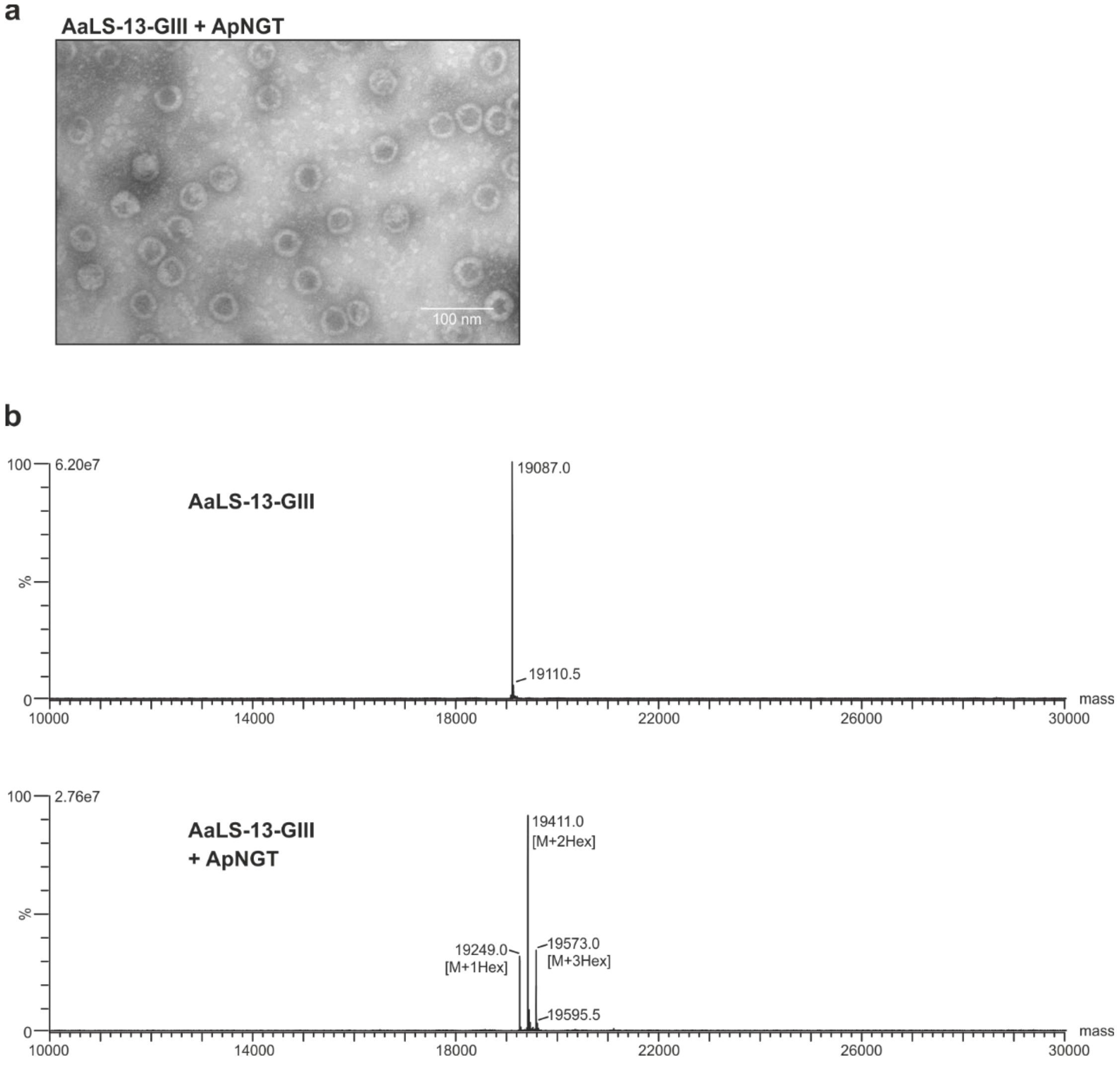

Supplementary Figure 9 – Glycosylation of AaLS-13 construct with three glycosites, AaLS-13-GIII. Three glycosylation sites were introduced into the surface-exposed C-terminus of AaLS-13. The construct was expressed in the absence and presence of the NGT, particles were purified, and characterized by transmission electron microscopy (**a**) and intact protein mass spectrometry (**b**).

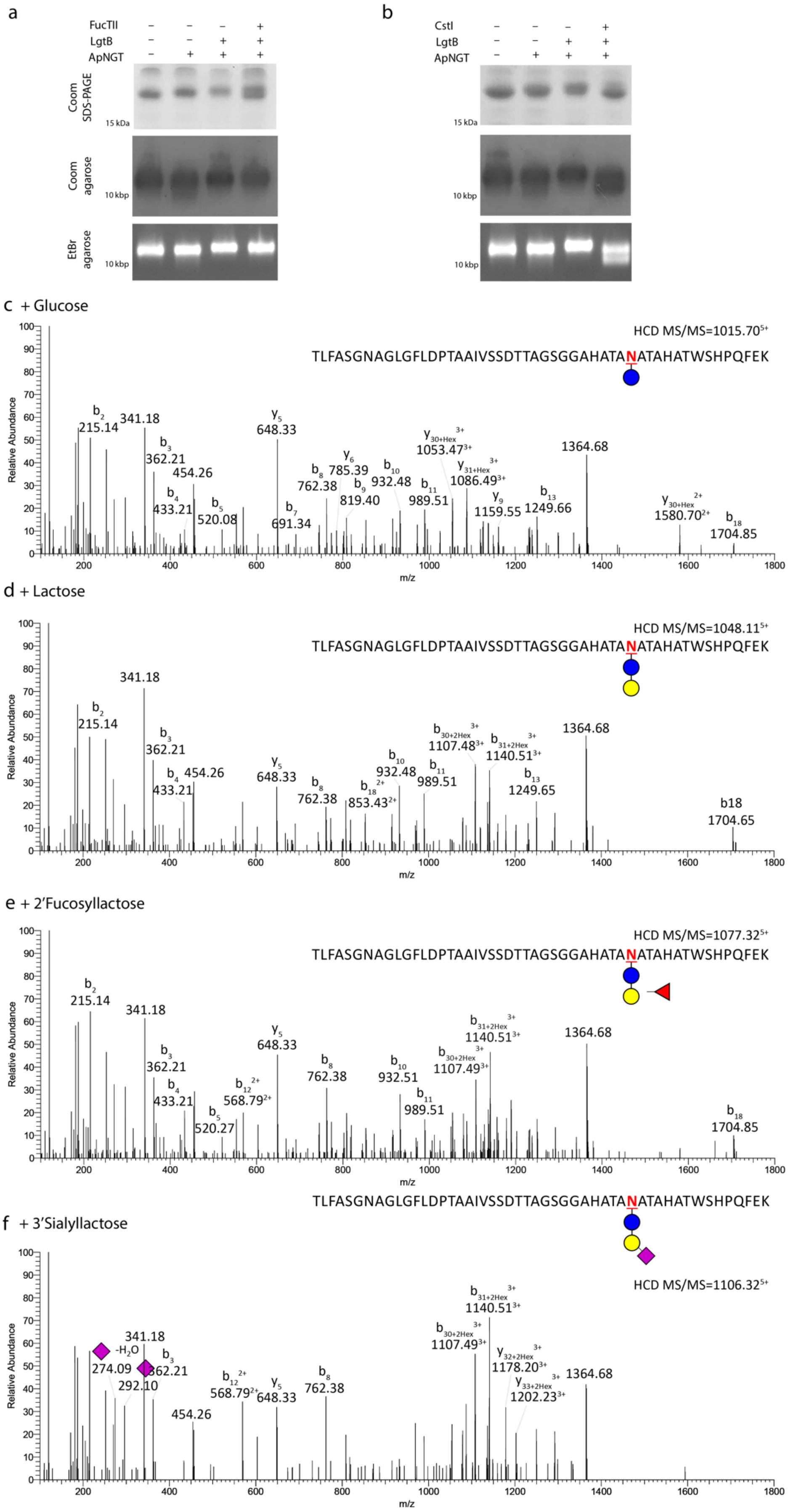
a
FucTII
LgtB
ApNGT
Coom SDS-PAGE
15 kDa
Coom agarose
10 kbp
EtBr agarose
10 kbp
b
CstI
LgtB
ApNGT
15 kDa
10 kbp
10 kbp
c + Glucose
HCD MS/MS=1015.70⁵⁺
TLFASGNAGLGFLDPTAAIVSSDTTAGSGGAHATANATAHATWSHPQFEK
Relative Abundance
m/z
d + Lactose
HCD MS/MS=1048.11⁵⁺
TLFASGNAGLGFLDPTAAIVSSDTTAGSGGAHATANATAHATWSHPQFEK
e + 2'Fucosyllactose
HCD MS/MS=1077.32⁵⁺
TLFASGNAGLGFLDPTAAIVSSDTTAGSGGAHATANATAHATWSHPQFEK
f + 3'Sialyllactose
TLFASGNAGLGFLDPTAAIVSSDTTAGSGGAHATANATAHATWSHPQFEK
HCD MS/MS=1106.32⁵⁺

Supplementary Figure 10 - Glycosylation of AP205 VLP. The AP205cp was co-expressed with the NGT, or with pathways for lactose, 2'-fucosyllactose, or 3'-sialyllactose. Purified particles were analyzed by denaturing PAGE with Coomassie (coom) staining (upper panel, **a** and **b**), and native agarose electrophoresis with ethidium bromide (EtBr) and Coomassie staining (lower panels, **a** and **b**). Glycosylation was evaluated by nano-LC-MS/MS of tryptic peptides (**c**-**f**). Displayed are HCD MS/MS spectra supporting the assigned structure of *m/z* species in **Figure 3d**.

Supplementary References

1 Naegeli, A. *et al.* Molecular analysis of an alternative N-glycosylation machinery by functional transfer from Actinobacillus pleuropneumoniae to Escherichia coli. *J Biol Chem* **289**, 2170-2179, (2014).

2 Lau, K. *et al.* Highly efficient chemoenzymatic synthesis of beta1-4-linked galactosides with promiscuous bacterial beta1-4-galactosyltransferases. *Chem Commun (Camb)* **46**, 6066-6068, (2010).

3 Blixt, O., van Die, I., Norberg, T. & van den Eijnden, D. H. High-level expression of the Neisseria meningitidis lgtA gene in Escherichia coli and characterization of the encoded N-acetylglucosaminyltransferase as a useful catalyst in the synthesis of GlcNAc beta 1-->3Gal and GalNAc beta 1-->3Gal linkages. *Glycobiology* **9**, 1061-1071 (1999).

4 Wang, G., Boulton, P. G., Chan, N. W., Palcic, M. M. & Taylor, D. E. Novel Helicobacter pylori alpha1,2-fucosyltransferase, a key enzyme in the synthesis of Lewis antigens. *Microbiology* **145**, 3245-3253 (1999).

5 Wang, G., Rasko, D. A., Sherburne, R. & Taylor, D. E. Molecular genetic basis for the variable expression of Lewis Y antigen in Helicobacter pylori: analysis of the alpha (1,2) fucosyltransferase gene. *Molecular microbiology* **31**, 1265-1274 (1999).

6 Baumgartner, F., Seitz, L., Sprenger, G. A. & Albermann, C. Construction of Escherichia coli strains with chromosomally integrated expression cassettes for the synthesis of 2'-fucosyllactose. *Microb Cell Fact* **12**, 40, (2013).

7 Wang, W. *et al.* Chemoenzymatic synthesis of GDP-L-fucose and the Lewis X glycan derivatives. *Proceedings of the National Academy of Sciences of the United States of America* **106**, 16096-16101, (2009).

8 Chiu, C. P. *et al.* Structural analysis of the alpha-2,3-sialyltransferase Cst-I from Campylobacter jejuni in apo and substrate-analogue bound forms. *Biochemistry* **46**, 7196-7204, (2007).

9 Chiu, C. P. *et al.* Structural analysis of the sialyltransferase CstII from Campylobacter jejuni in complex with a substrate analog. *Nat Struct Mol Biol* **11**, 163-170, (2004).

10 Keys, T. G. *et al.* Engineering the product profile of a polysialyltransferase. *Nature chemical biology* **10**, 437-442, (2014).

11 Peterson, D. C., Arakere, G., Vionnet, J., McCarthy, P. C. & Vann, W. F. Characterization and acceptor preference of a soluble meningococcal group C polysialyltransferase. *J Bacteriol* **193**, 1576-1582, (2011).

12 Florea, M. *et al.* Engineering control of bacterial cellulose production using a genetic toolkit and a new cellulose-producing strain. *Proceedings of the National Academy of Sciences of the United States of America* **113**, E3431-3440, (2016).

13 Keys, T. G. *et al.* A biosynthetic route for polysialylating proteins in Escherichia coli. *Metab Eng* **44**, 293-301, (2017).
