# Supplementary material for: Cytoplasmic glycoengineering enables biosynthesis of nanoscale glycoprotein assemblies
Source: Nat Commun. 2019 Nov 27;10:5403. doi: 10.1038/s41467-019-13283-2 (PMC6881330; doi:10.1038/s41467-019-13283-2)
Supplement: Supplementary file 2 — Reporting Summary [file 41467_2019_13283_MOESM2_ESM.pdf]

nature research

Corresponding author(s): Tim Keys

Last updated by author(s): 25.10.2019

Reporting Summary

Nature Research wishes to improve the reproducibility of the work that we publish. This form provides structure for consistency and transparency in reporting. For further information on Nature Research policies, see Authors & Referees and the Editorial Policy Checklist.

Statistics

For all statistical analyses, confirm that the following items are present in the figure legend, table legend, main text, or Methods section.

n/a	Confirmed	
☐	☒	The exact sample size (*n*) for each experimental group/condition, given as a discrete number and unit of measurement
☐	☒	A statement on whether measurements were taken from distinct samples or whether the same sample was measured repeatedly
☒	☐	The statistical test(s) used AND whether they are one- or two-sided<br>*Only common tests should be described solely by name; describe more complex techniques in the Methods section.*
☒	☐	A description of all covariates tested
☒	☐	A description of any assumptions or corrections, such as tests of normality and adjustment for multiple comparisons
☐	☒	A full description of the statistical parameters including central tendency (e.g. means) or other basic estimates (e.g. regression coefficient) AND variation (e.g. standard deviation) or associated estimates of uncertainty (e.g. confidence intervals)
☒	☐	For null hypothesis testing, the test statistic (e.g. *F*, *t*, *r*) with confidence intervals, effect sizes, degrees of freedom and *P* value noted<br>*Give P values as exact values whenever suitable.*
☒	☐	For Bayesian analysis, information on the choice of priors and Markov chain Monte Carlo settings
☒	☐	For hierarchical and complex designs, identification of the appropriate level for tests and full reporting of outcomes
☒	☐	Estimates of effect sizes (e.g. Cohen's *d*, Pearson's *r*), indicating how they were calculated

Our web collection on statistics for biologists contains articles on many of the points above.

Software and code

Policy information about availability of computer code

Data collection	XCalibur 4.1 was used for recording of LC-MS data. Chromeleon 7.2 was used for collection of UPLC data.
Data analysis	Byonic 3.1 was used for analysis of LC-MS data. Chromeleon 7.2 was used for analysis of UPLC data.

For manuscripts utilizing custom algorithms or software that are central to the research but not yet described in published literature, software must be made available to editors/reviewers. We strongly encourage code deposition in a community repository (e.g. GitHub). See the Nature Research guidelines for submitting code & software for further information.

Data

Policy information about availability of data

All manuscripts must include a data availability statement. This statement should provide the following information, where applicable:

- Accession codes, unique identifiers, or web links for publicly available datasets
- A list of figures that have associated raw data
- A description of any restrictions on data availability

All data associated with this manuscript will be made freely available upon request. Whenever applicable, accession codes have been provided in the manuscript to unequivocally identify DNA or protein sequences used.

Field-specific reporting

Please select the one below that is the best fit for your research. If you are not sure, read the appropriate sections before making your selection.

☒ Life sciences ☐ Behavioural & social sciences ☐ Ecological, evolutionary & environmental sciences

For a reference copy of the document with all sections, see nature.com/documents/nr-reporting-summary-flat.pdf

Life sciences study design

All studies must disclose on these points even when the disclosure is negative.

Sample size	All glycoprotein samples were produced and analyzed in at least three independent batches. All samples were subjected to SDS-PAGE and MS analysis to confirm consistent structure and quantity of the product. In each case the data from one representative sample was annotated and presented in the manuscript.
Data exclusions	No data were excluded.
Replication	All glycoprotein samples were produced and analyzed in at least three independent cultures. All attempts at replication were successful.
Randomization	No randomization was carried out. The experimenter has no subjective influence on the outcome of PAGE, MS, UPLC, or TEM analyses.
Blinding	The experimenter was not blinded to samples. The experimenter has no subjective influence on the outcome of PAGE, MS, UPLC, or TEM analyses.

Reporting for specific materials, systems and methods

We require information from authors about some types of materials, experimental systems and methods used in many studies. Here, indicate whether each material, system or method listed is relevant to your study. If you are not sure if a list item applies to your research, read the appropriate section before selecting a response.

Materials & experimental systems

n/a	Involved in the study	
☐	☒	Antibodies
☒	☐	Eukaryotic cell lines
☒	☐	Palaeontology
☐	☒	Animals and other organisms
☒	☐	Human research participants
☒	☐	Clinical data

Methods

n/a	Involved in the study	
☒	☐	ChIP-seq
☒	☐	Flow cytometry
☒	☐	MRI-based neuroimaging

Antibodies

Antibodies used	mouse anti-His4 IgG (Qiagen, 34670); goat anti-mouse IgG-HRP (Santa Cruz Biotech., sc-2005); anti-PSA IgG2a (Absolute Antibody, 735, Ab00240-2.0); rabbit anti-NmC (Neisseria meningitidis serogroup C) antiserum (BD Biosciences, 222301); goat anti-rabbit IgG-HRP (Santa Cruz Biotech., sc-2004); human anti-N-Glc serum MS14 (gift from Prof Anna Maria Papini).
Validation	Commercial antibodies were validated by the vendor. The MS14 serum was validated in earlier work (Naegeli etal., JBC, 2014).

Animals and other organisms

Policy information about studies involving animals; ARRIVE guidelines recommended for reporting animal research

Laboratory animals	The study did not involve animals. The study involved common laboratory strains of Escherichia coli.
Wild animals	No wild animals were involved in the study
Field-collected samples	No field samples were collected.
Ethics oversight	Ethical oversight was not required for the microbial cutivation carried out in this study.

Note that full information on the approval of the study protocol must also be provided in the manuscript.
