# Supplementary material for: Cytoplasmic glycoengineering enables biosynthesis of nanoscale glycoprotein assemblies
Source: Nat Commun. 2019 Nov 27;10:5403. doi: 10.1038/s41467-019-13283-2 (PMC6881330; doi:10.1038/s41467-019-13283-2)
Supplement: Supplementary file 4 — Source Data [file 41467_2019_13283_MOESM4_ESM.zip › HPLC_data_GFP-SiaLac.pdf]

Chromatogram and Results

Injection Details

Injection Name:	**3ug_GFP1045+pHT081_I**	*Run Time (min):*	**26.00**
Vial Number:	**BH10**	*Injection Volume:*	**50.00**
Injection Type:	**Unknown**	*Channel:*	**Emission_1**
Calibration Level:		*Wavelength:*	**n.a.**
Instrument Method:	**ProPacSAX__GFP-SiaLac**	*Bandwidth:*	**n.a.**
Processing Method:	**GFP-SiaLac**	*Dilution Factor:*	**1.0000**
Injection Date/Time:	**12.Aug.19 10:04**	*Sample Weight:*	**1.0000**

Chromatogram

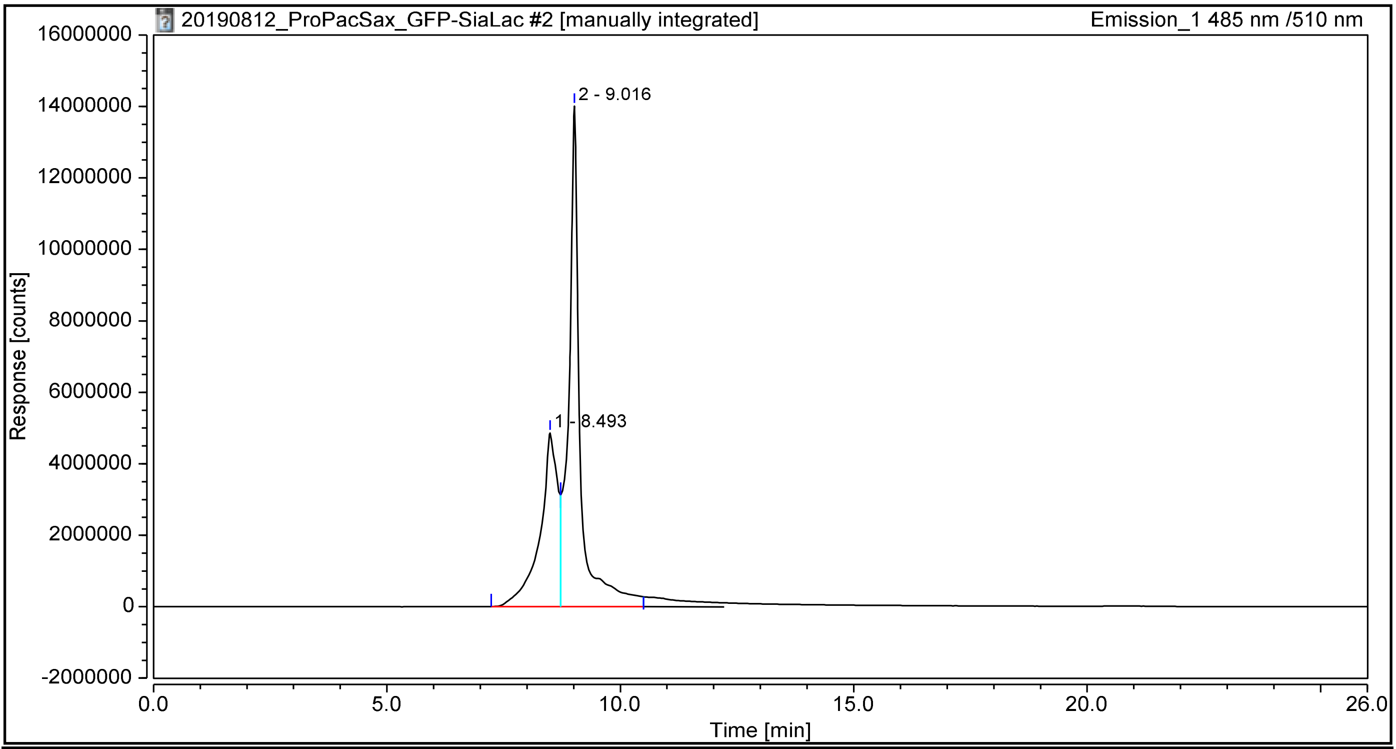

Integration Results

No.	Peak Name	Retention Time min	Area counts*min	Height counts	Relative Area %	Relative Height %	Amount n.a.
1		8.493	2128692.226	4857565.351	34.80	25.75	n.a.
2		9.016	3987971.648	14007591.037	65.20	74.25	n.a.
**Total:**			**6116663.874**	**18865156.388**	**100.00**	**100.00**	

Chromatogram and Results

Injection Details

Injection Name:	**3ug_GFP1045+pHT081_II**	*Run Time (min):*	**26.00**
Vial Number:	**BH11**	*Injection Volume:*	**50.00**
Injection Type:	**Unknown**	*Channel:*	**Emission_1**
Calibration Level:		*Wavelength:*	**n.a.**
Instrument Method:	**ProPacSAX__GFP-SiaLac**	*Bandwidth:*	**n.a.**
Processing Method:	**GFP-SiaLac**	*Dilution Factor:*	**1.0000**
Injection Date/Time:	**12.Aug.19 10:31**	*Sample Weight:*	**1.0000**

Chromatogram

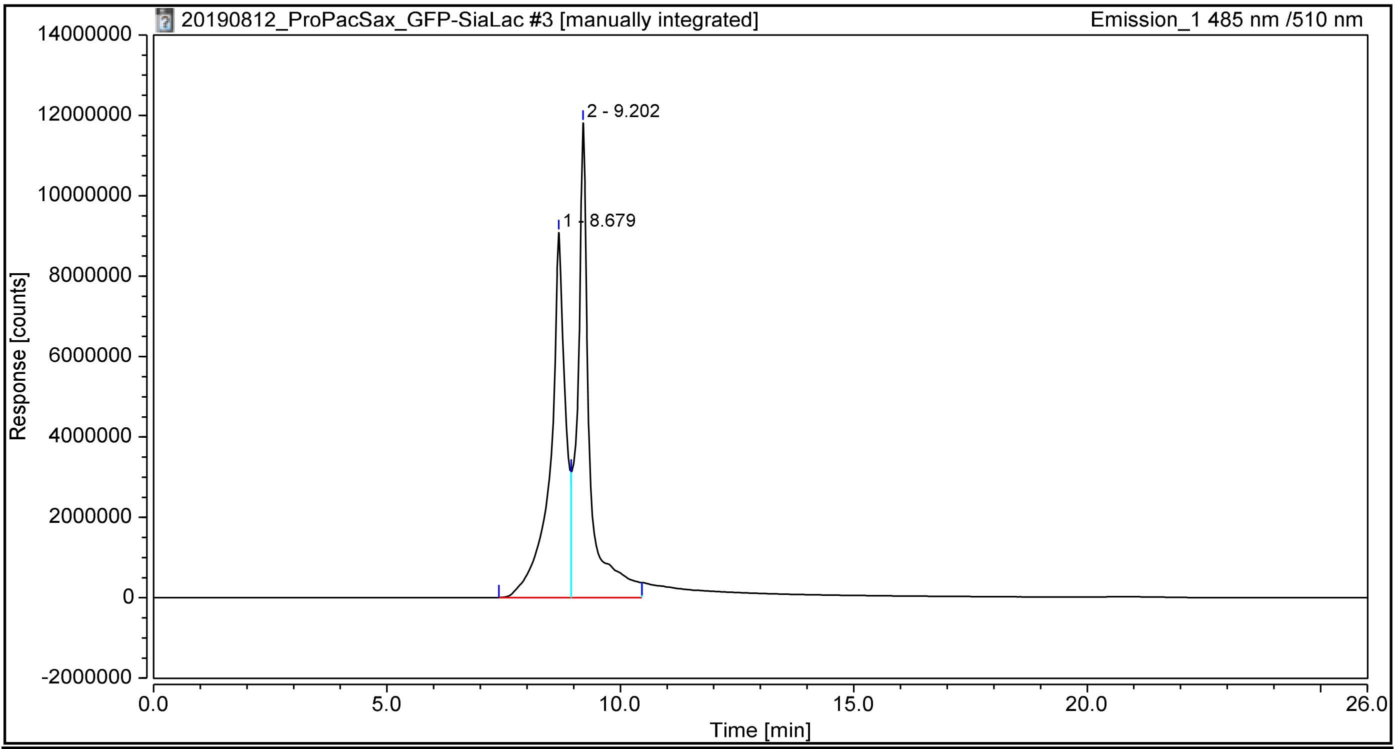

Integration Results

No.	Peak Name	Retention Time min	Area counts*min	Height counts	Relative Area %	Relative Height %	Amount n.a.
1		8.679	3354837.138	9082517.889	49.13	43.49	n.a.
2		9.202	3474041.340	11802470.968	50.87	56.51	n.a.
**Total:**			**6828878.479**	**20884988.857**	**100.00**	**100.00**	

Chromatogram and Results

Injection Details

Injection Name:	**3ug_GFP1045+pHT081_III**	*Run Time (min):*	**26.00**
Vial Number:	**BH12**	*Injection Volume:*	**50.00**
Injection Type:	**Unknown**	*Channel:*	**Emission_1**
Calibration Level:		*Wavelength:*	**n.a.**
Instrument Method:	**ProPacSAX__GFP-SiaLac**	*Bandwidth:*	**n.a.**
Processing Method:	**GFP-SiaLac**	*Dilution Factor:*	**1.0000**
Injection Date/Time:	**12.Aug.19 10:58**	*Sample Weight:*	**1.0000**

Chromatogram

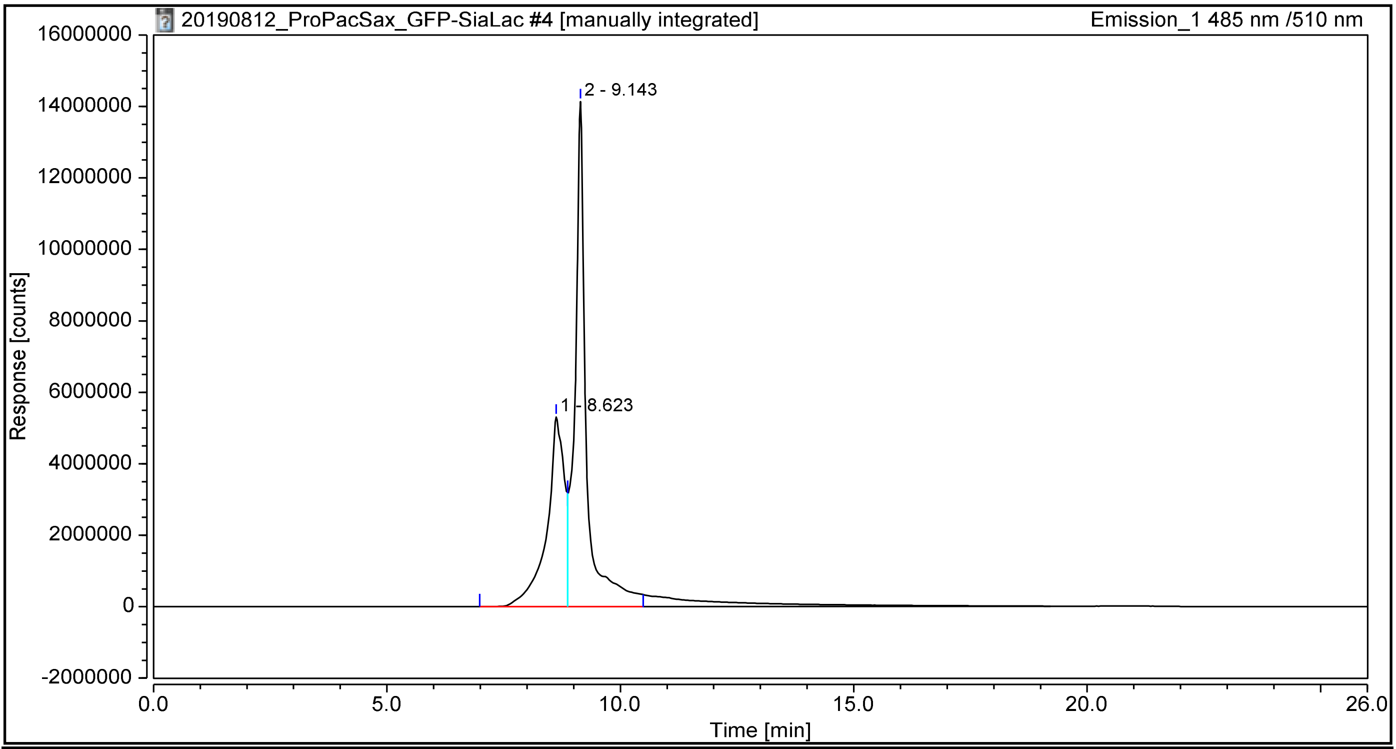

Integration Results

No.	Peak Name	Retention Time min	Area counts*min	Height counts	Relative Area %	Relative Height %	Amount n.a.
1		8.623	2289742.367	5310138.725	36.54	27.31	n.a.
2		9.143	3977188.831	14132178.403	63.46	72.69	n.a.
**Total:**			**6266931.198**	**19442317.128**	**100.00**	**100.00**	

Chromatogram and Results

Injection Details

Injection Name:	**3ug_GFP1045_unmodified**	*Run Time (min):*	**26.00**
Vial Number:	**BG10**	*Injection Volume:*	**50.00**
Injection Type:	**Unknown**	*Channel:*	**Emission_1**
Calibration Level:		*Wavelength:*	**n.a.**
Instrument Method:	**ProPacSAX__GFP-SiaLac_OSP**	*Bandwidth:*	**n.a.**
Processing Method:	**GFP-SiaLac**	*Dilution Factor:*	**1.0000**
Injection Date/Time:	**12.Aug.19 11:26**	*Sample Weight:*	**1.0000**

Chromatogram

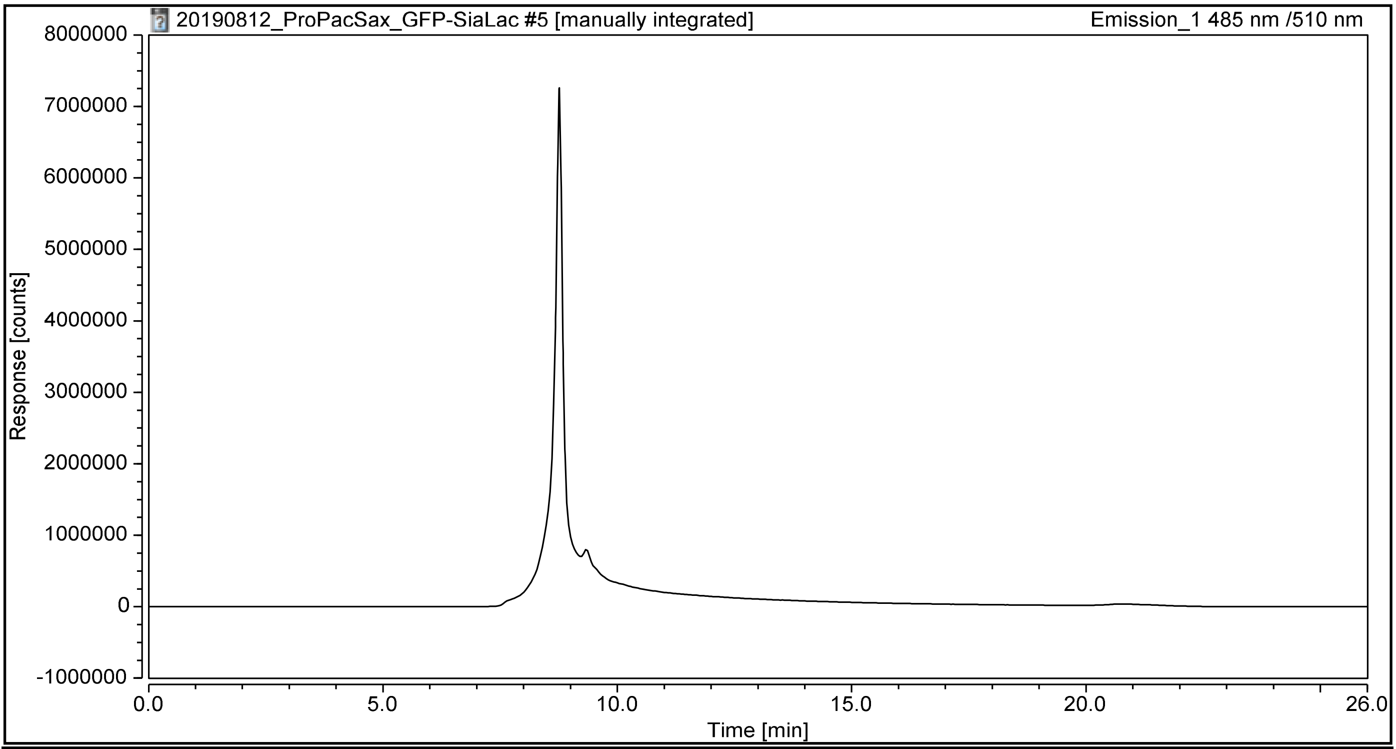

Integration Results

No.	Peak Name	Retention Time min	Area counts*min	Height counts	Relative Area %	Relative Height %	Amount n.a.
**Total:**			**0.000**	**0.000**	**0.00**	**0.00**	

Chromatogram and Results

Injection Details

Injection Name:	**3ug_GFP1045-Glc**	*Run Time (min):*	**26.00**
Vial Number:	**BG11**	*Injection Volume:*	**10.00**
Injection Type:	**Unknown**	*Channel:*	**Emission_1**
Calibration Level:		*Wavelength:*	**n.a.**
Instrument Method:	**ProPacSAX__GFP-SiaLac_OSP**	*Bandwidth:*	**n.a.**
Processing Method:	**GFP-SiaLac**	*Dilution Factor:*	**1.0000**
Injection Date/Time:	**12.Aug.19 11:52**	*Sample Weight:*	**1.0000**

Chromatogram

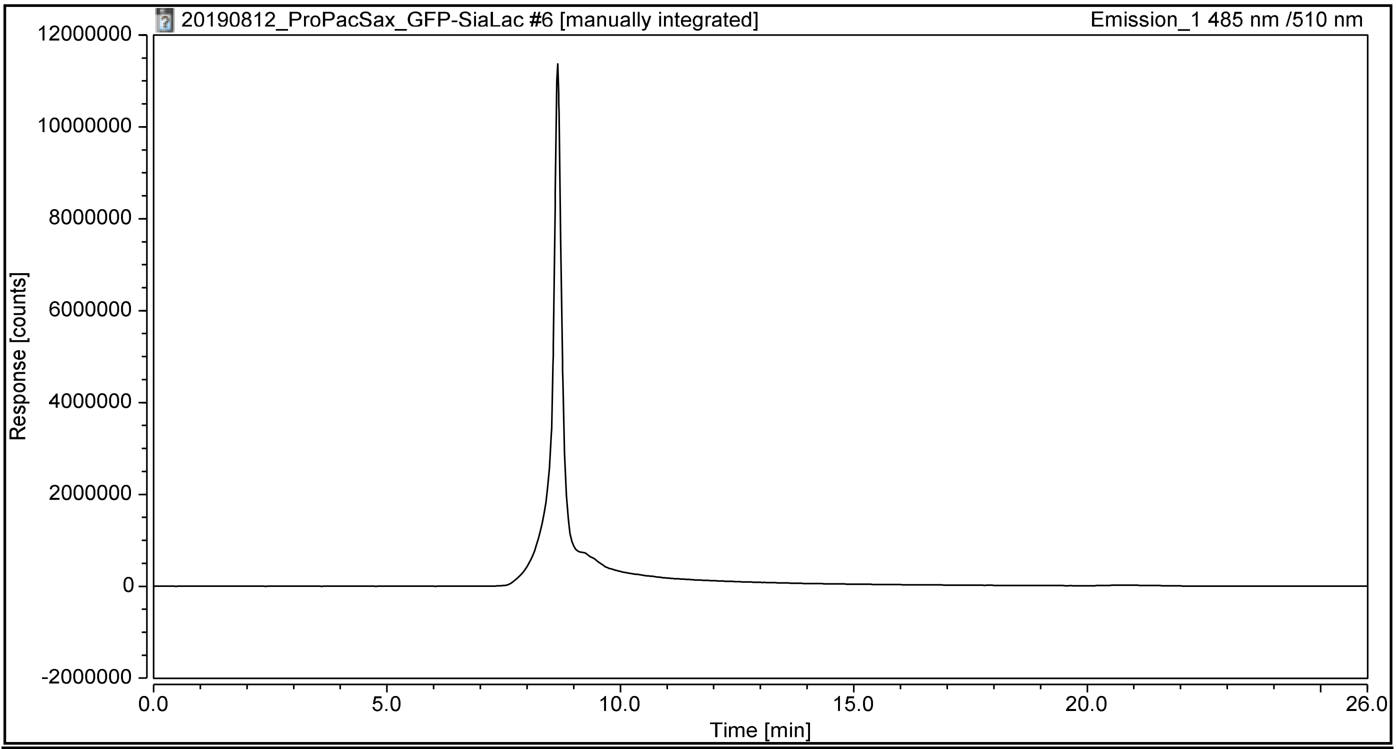

Integration Results

No.	Peak Name	Retention Time min	Area counts*min	Height counts	Relative Area %	Relative Height %	Amount n.a.
**Total:**			**0.000**	**0.000**	**0.00**	**0.00**	

Chromatogram and Results

Injection Details

Injection Name:	**3ug_GFP1045-Lac**	*Run Time (min):*	**26.00**
Vial Number:	**BG12**	*Injection Volume:*	**10.00**
Injection Type:	**Unknown**	*Channel:*	**Emission_1**
Calibration Level:		*Wavelength:*	**n.a.**
Instrument Method:	**ProPacSAX__GFP-SiaLac_OSP**	*Bandwidth:*	**n.a.**
Processing Method:	**GFP-SiaLac**	*Dilution Factor:*	**1.0000**
Injection Date/Time:	**12.Aug.19 12:18**	*Sample Weight:*	**1.0000**

Chromatogram

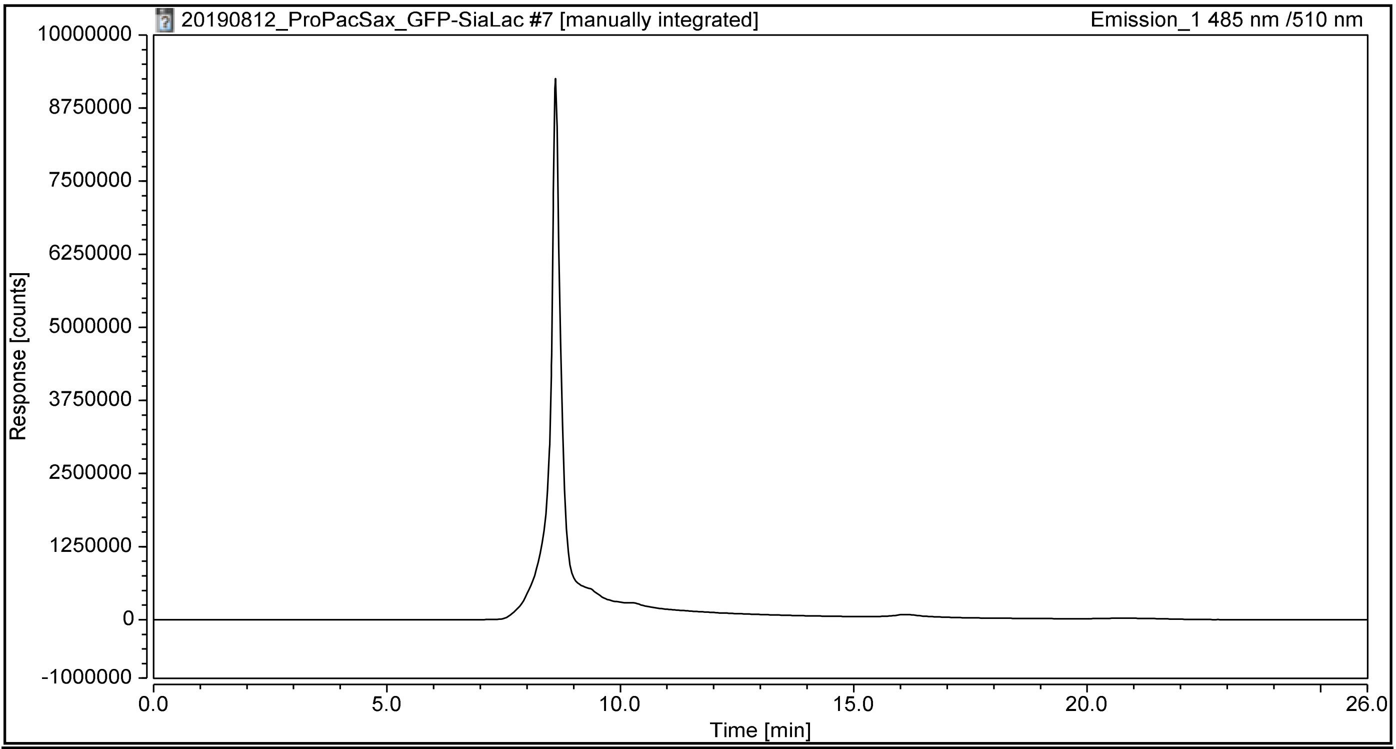

Integration Results

No.	Peak Name	Retention Time<br>min	Area<br>counts*min	Height<br>counts	Relative Area<br>%	Relative Height<br>%	Amount<br>n.a.
**Total:**			**0.000**	**0.000**	**0.00**	**0.00**	
