# Supplementary material for: Cytoplasmic glycoengineering enables biosynthesis of nanoscale glycoprotein assemblies
Source: Nat Commun. 2019 Nov 27;10:5403. doi: 10.1038/s41467-019-13283-2 (PMC6881330; doi:10.1038/s41467-019-13283-2)
Supplement: Supplementary file 4 — Source Data [file 41467_2019_13283_MOESM4_ESM.zip › MS_data_GFP-Glc.pdf]

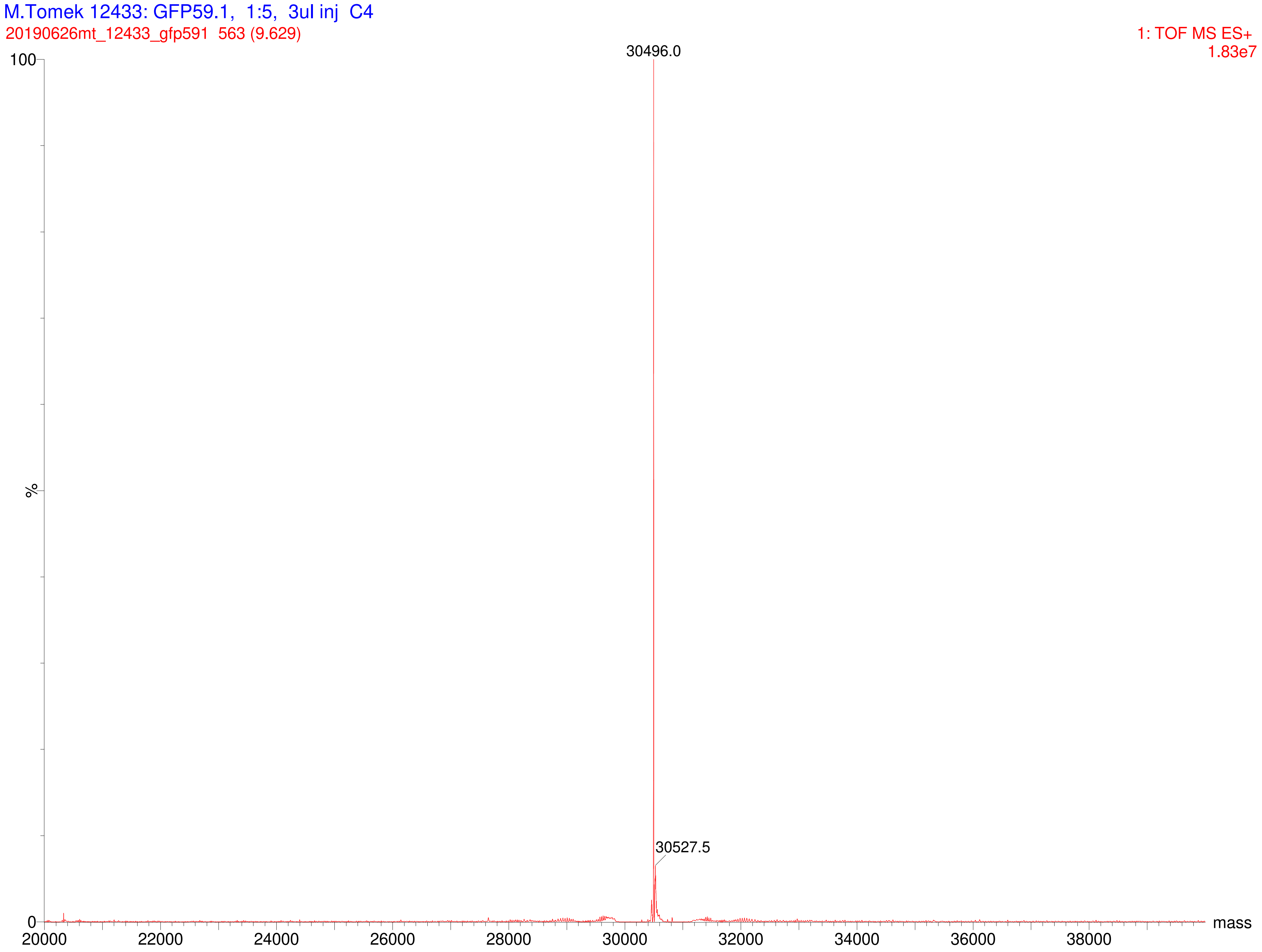

M.Tomek 12433: GFP59.1, 1:5, 3ul inj C4
20190626mt_12433_gfp591 563 (9.629)
1: TOF MS ES+
1.83e7
30496.0
30527.5
100
%
0
20000
22000
24000
26000
28000
30000
32000
34000
36000
38000
mass

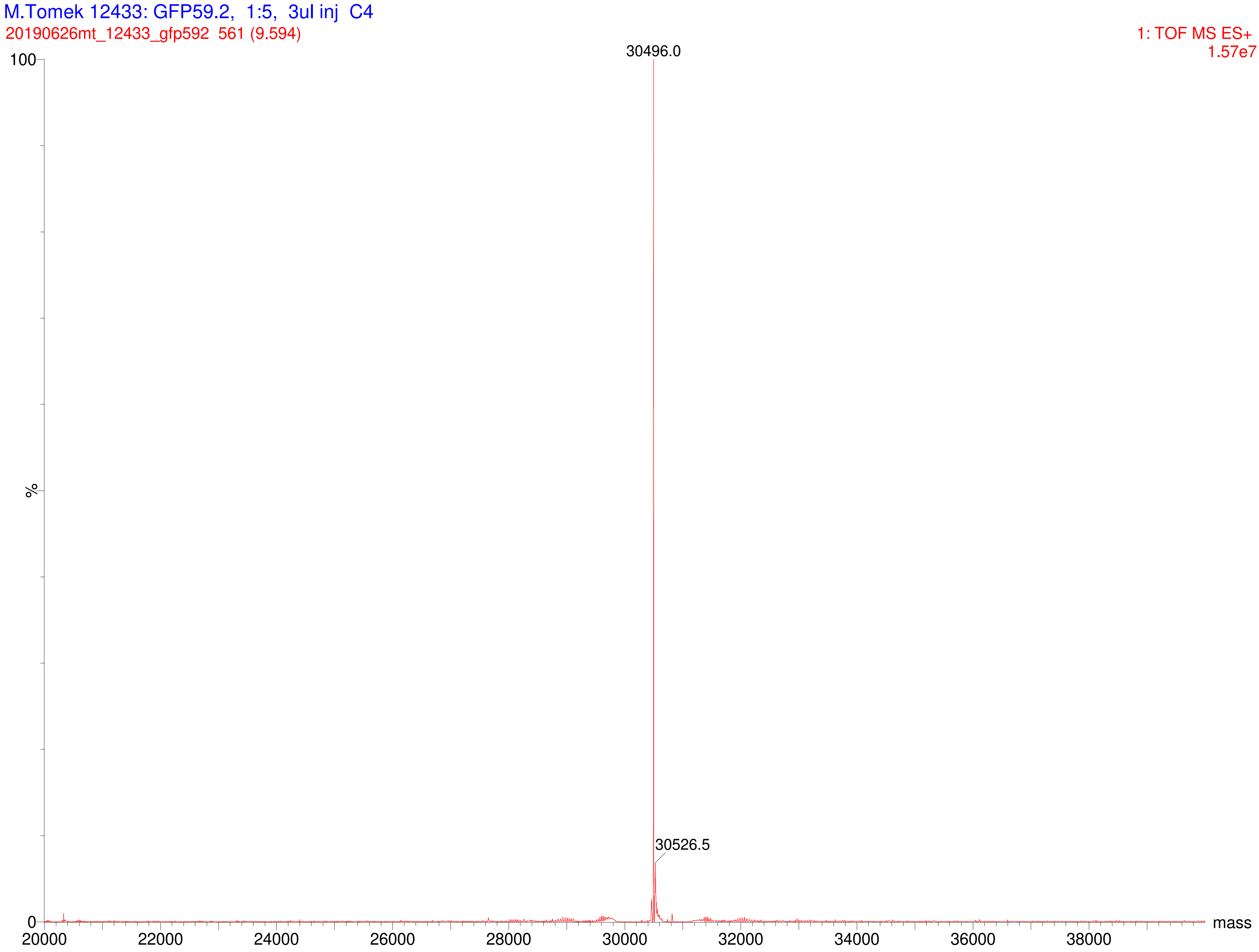

M.Tomek 12433: GFP59.2, 1:5, 3ul inj C4
20190626mt_12433_gfp592 561 (9.594)
1: TOF MS ES+
1.57e7
30496.0
30526.5
100
%
0
20000
22000
24000
26000
28000
30000
32000
34000
36000
38000
mass

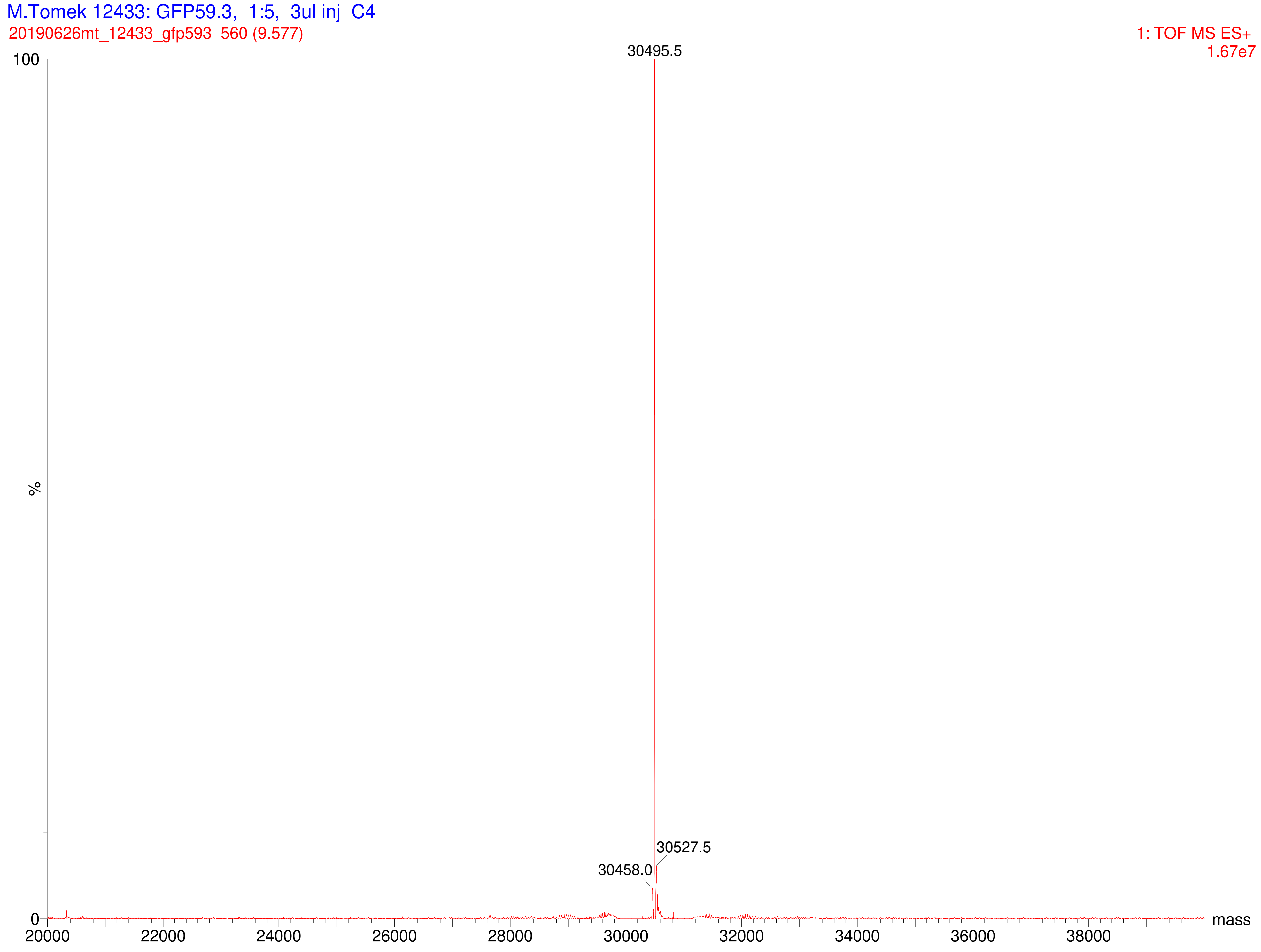
M.Tomek 12433: GFP59.3, 1:5, 3ul inj C4
20190626mt_12433_gfp593 560 (9.577)
1: TOF MS ES+
1.67e7
30495.5
30527.5
30458.0
100
%
0
mass
20000
22000
24000
26000
28000
30000
32000
34000
36000
38000
