# Supplementary material for: Cytoplasmic glycoengineering enables biosynthesis of nanoscale glycoprotein assemblies
Source: Nat Commun. 2019 Nov 27;10:5403. doi: 10.1038/s41467-019-13283-2 (PMC6881330; doi:10.1038/s41467-019-13283-2)
Supplement: Supplementary file 4 — Source Data [file 41467_2019_13283_MOESM4_ESM.zip › MS_data_GFP-Lac.pdf]

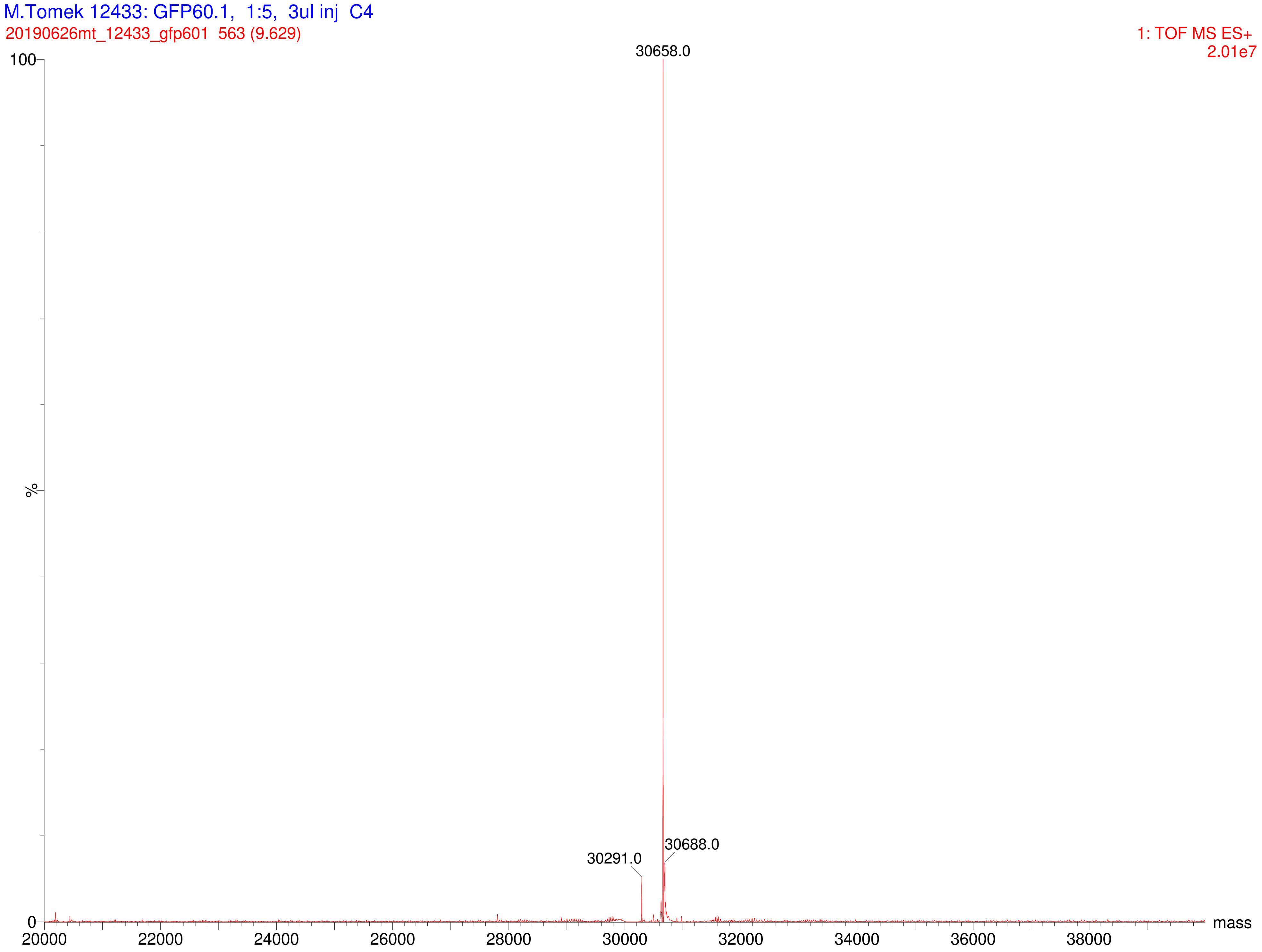

M.Tomek 12433: GFP60.1, 1:5, 3ul inj C4
20190626mt_12433_gfp601 563 (9.629)
1: TOF MS ES+
2.01e7
30658.0
100
%
30688.0
30291.0
0
20000
22000
24000
26000
28000
30000
32000
34000
36000
38000
mass

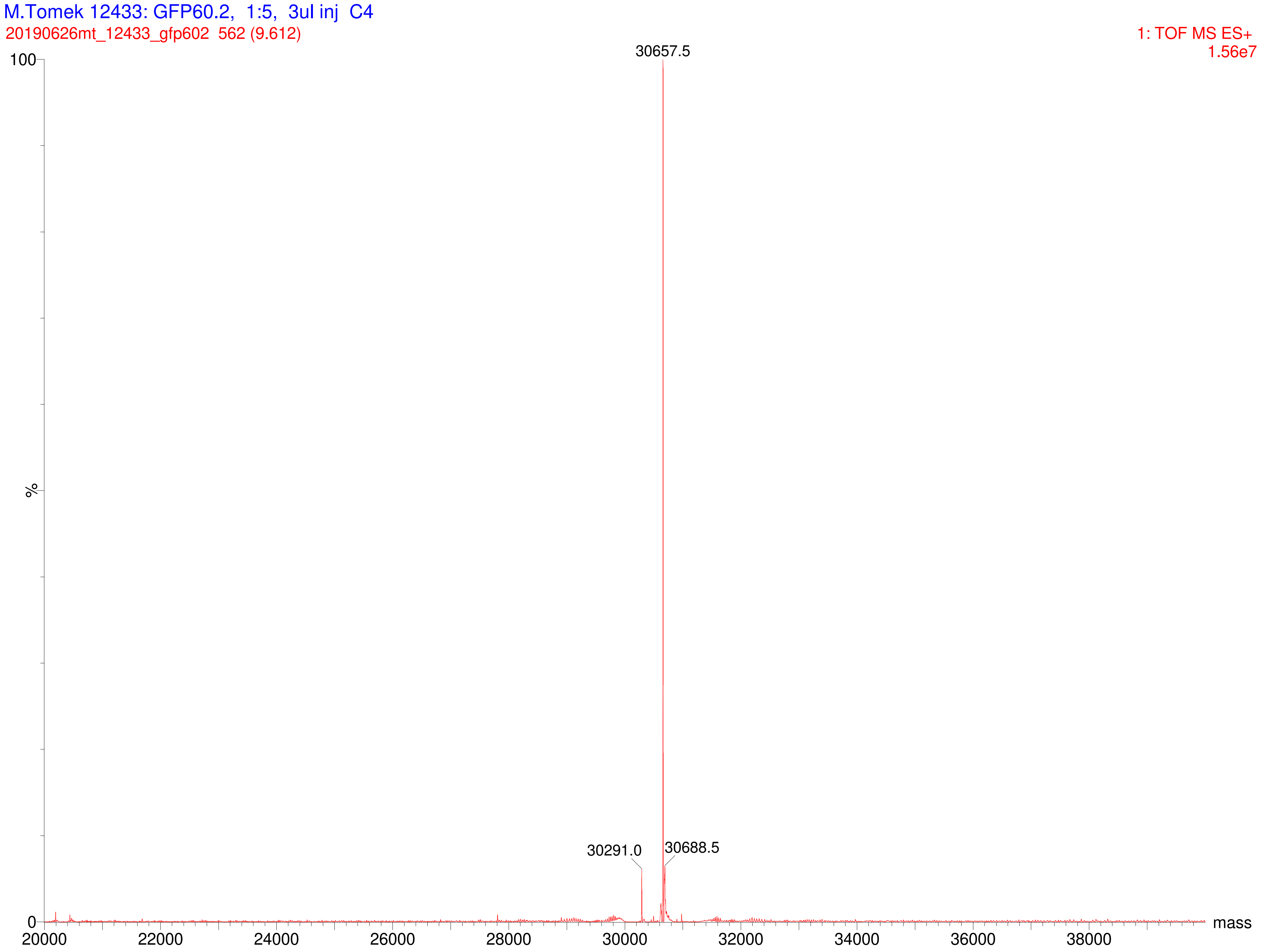
M.Tomek 12433: GFP60.2, 1:5, 3ul inj C4
20190626mt_12433_gfp602 562 (9.612)
1: TOF MS ES+
1.56e7
30657.5
30291.0
30688.5
100
%
0
20000
22000
24000
26000
28000
30000
32000
34000
36000
38000
mass

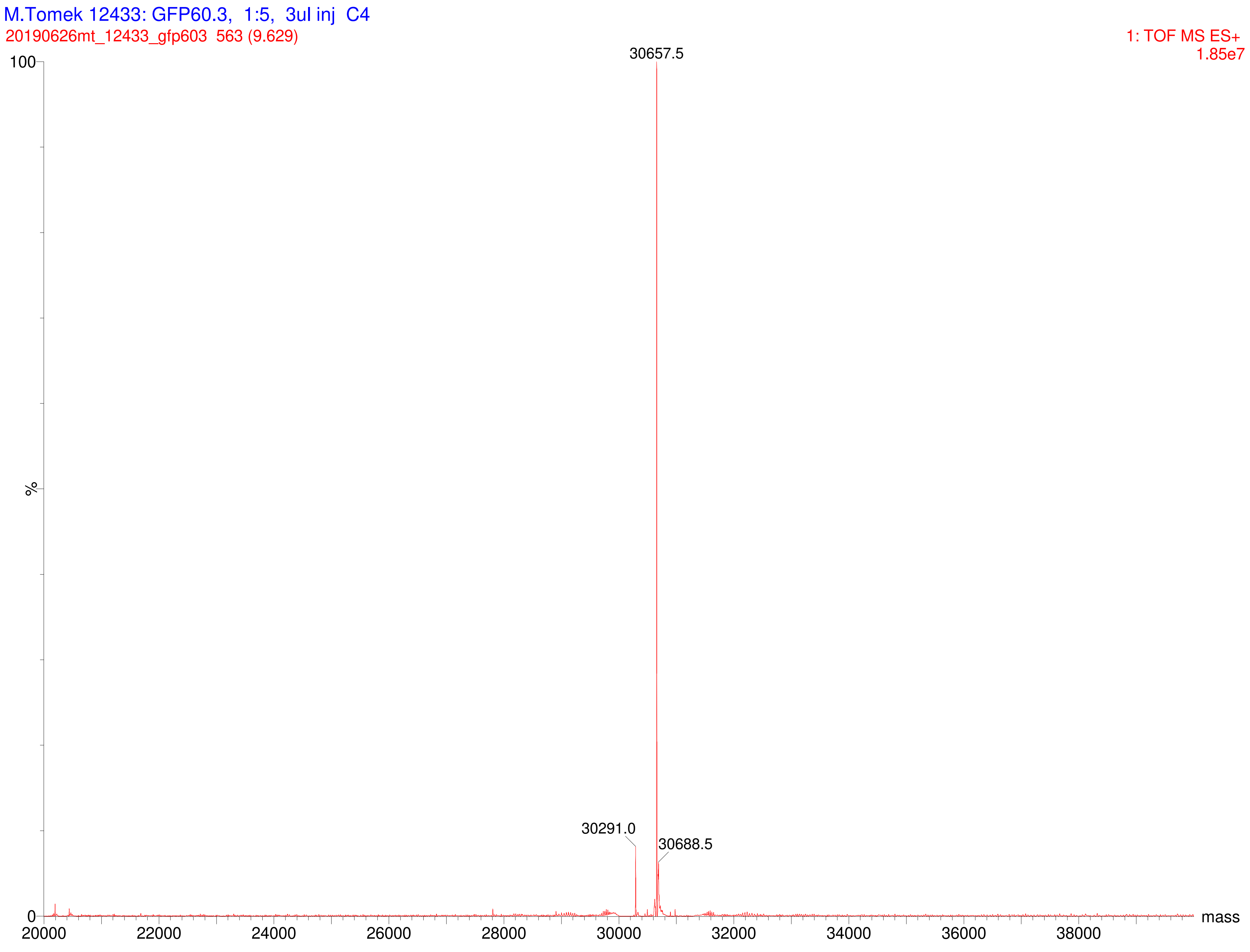
M.Tomek 12433: GFP60.3, 1:5, 3ul inj C4
20190626mt_12433_gfp603 563 (9.629)
1: TOF MS ES+
1.85e7
30657.5
30291.0
30688.5
100
%
0
20000
22000
24000
26000
28000
30000
32000
34000
36000
38000
mass
