# Supplementary figures and images for: Cytoplasmic glycoengineering enables biosynthesis of nanoscale glycoprotein assemblies
Source: Nat Commun. 2019 Nov 27;10:5403. doi: 10.1038/s41467-019-13283-2 (PMC6881330; doi:10.1038/s41467-019-13283-2)

M. Tomek 12464: dil. 1:10: 1 ul C4 ZT: 10.59.1

20190903MT\_12464\_2\_10591 194 (3.297)

TOF MS ES+  
1.61e8

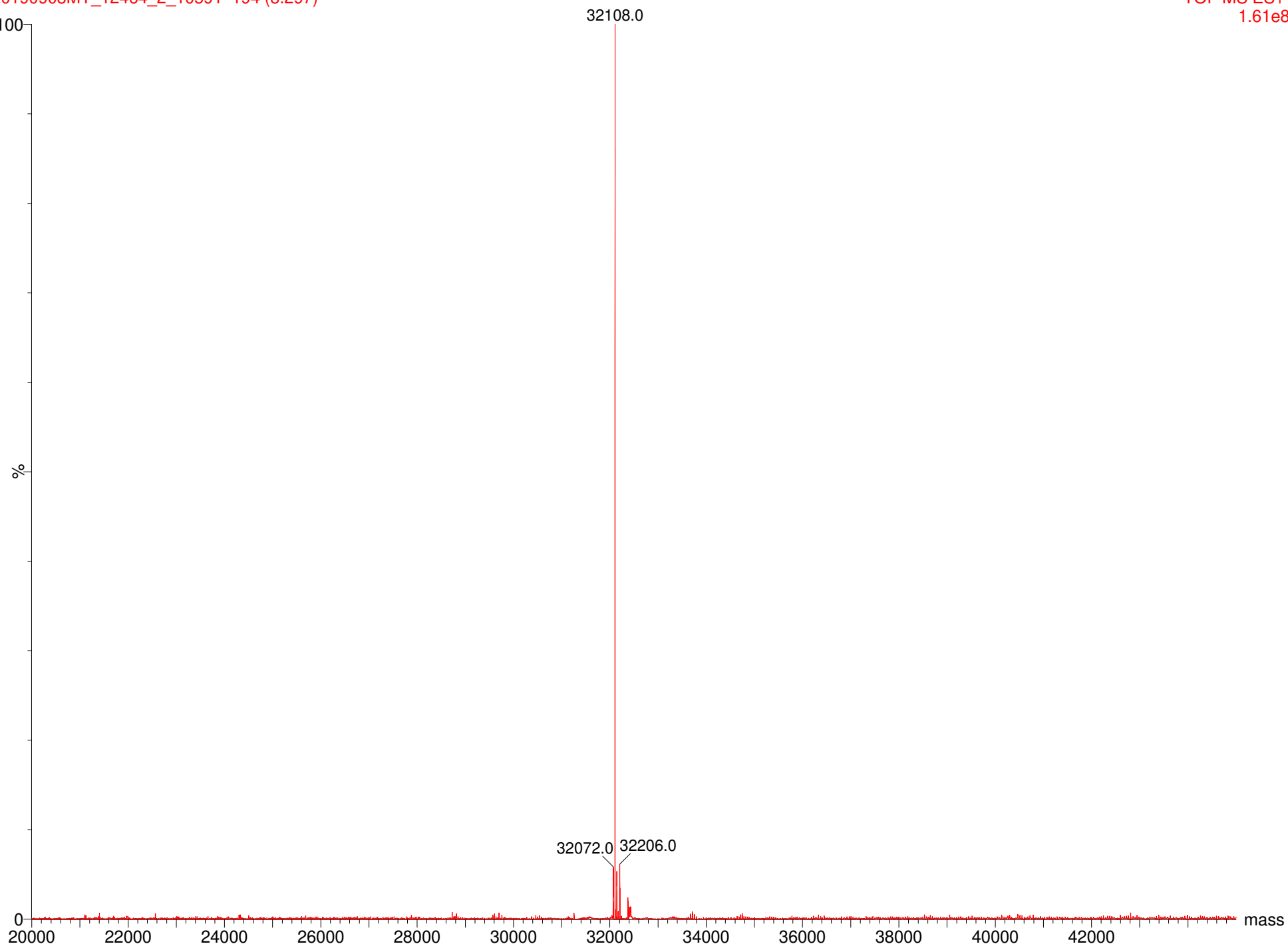

M. Tomek 12464: dil. 1:10: 2 ul C4 ZT: 10.59.2

20190903MT\_12464\_10592 128 (2.180)

TOF MS ES+  
8.91e7

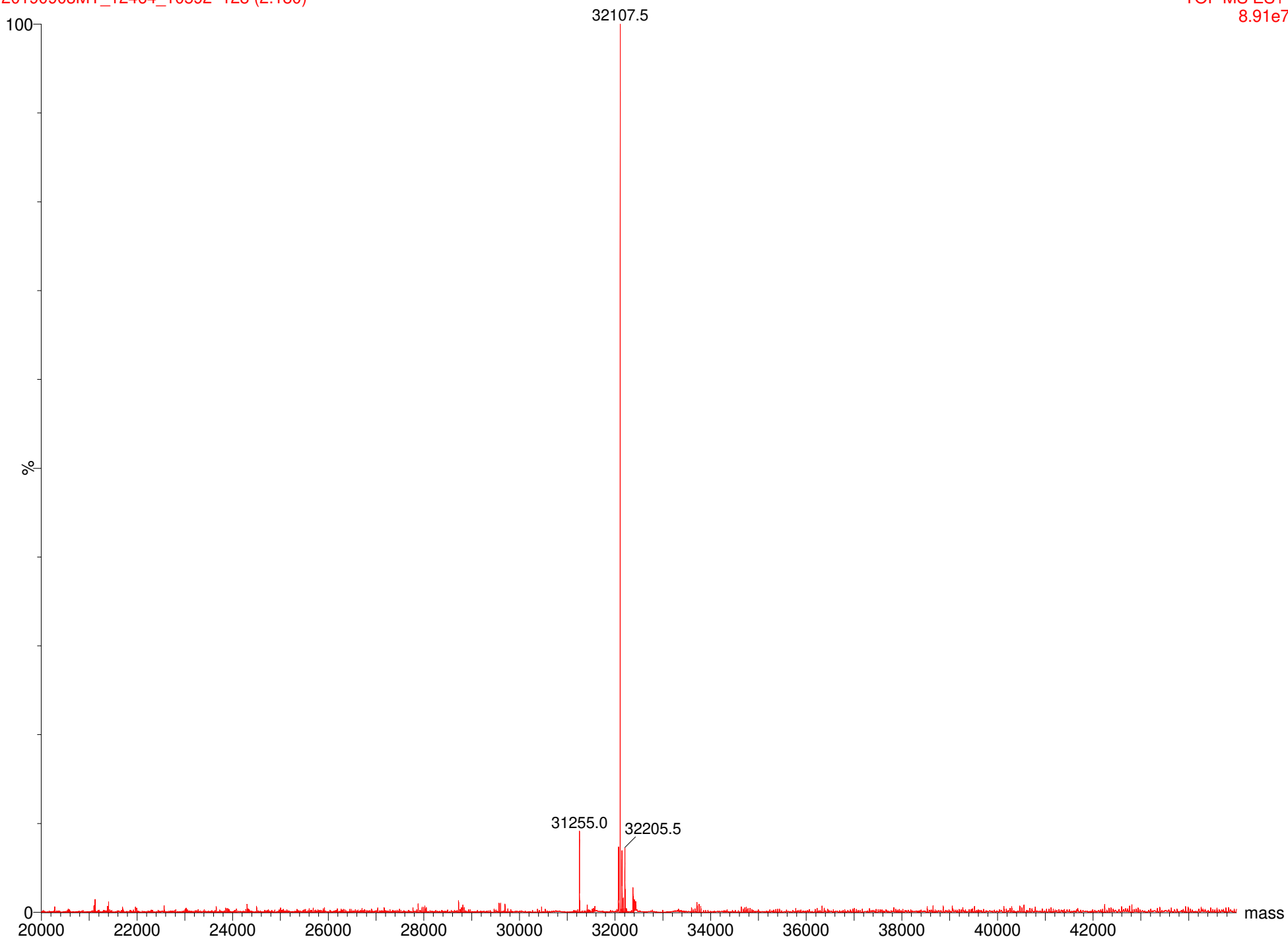

M. Tomek 12464: dil. 1:10: 1 ul C4 ZT: 10.59.3

20190903MT\_12464\_10593 145 (2.468)

TOF MS ES+  
4.89e7

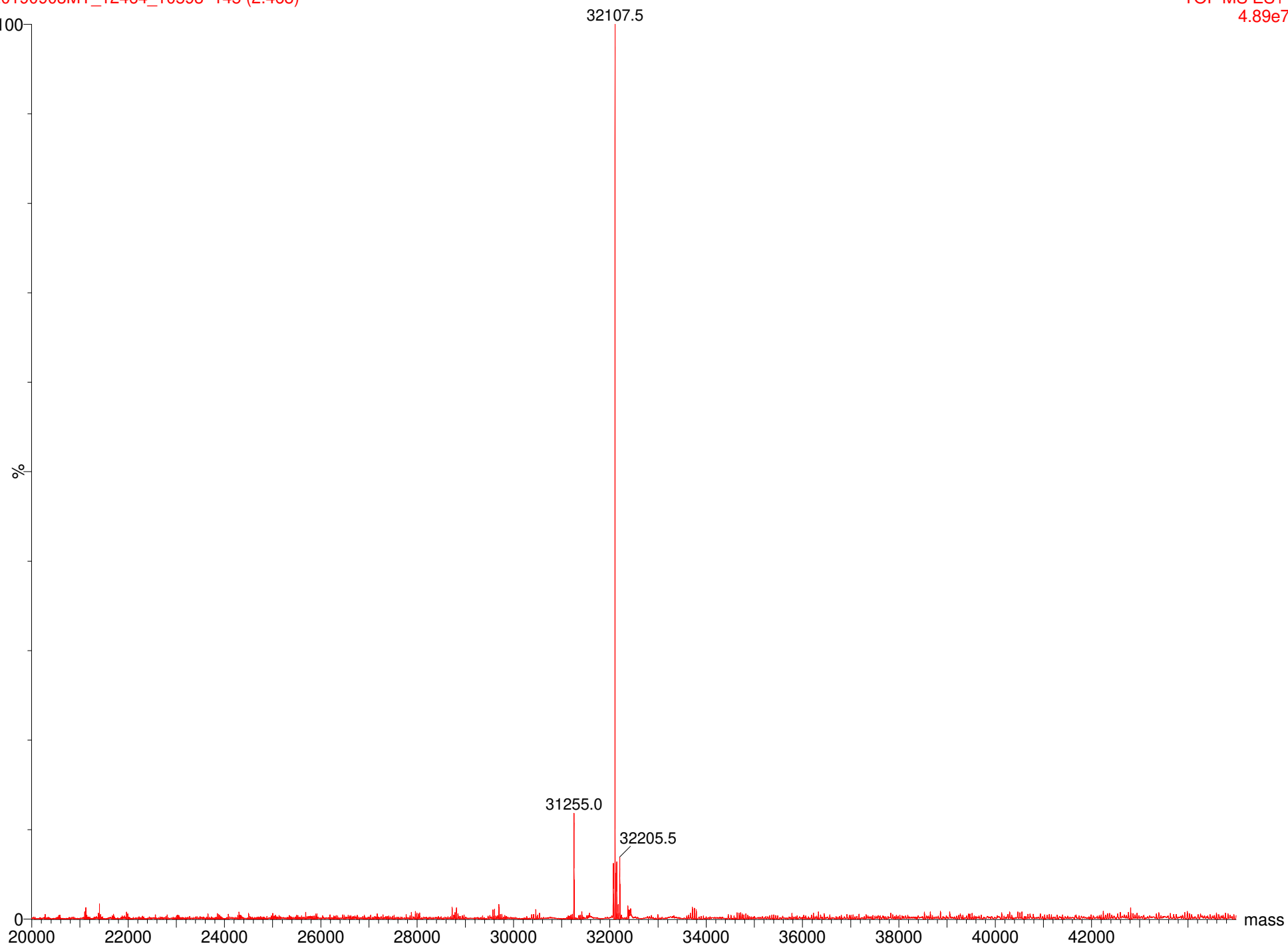

Supplement: Supplementary file 4 — Source Data [file 41467_2019_13283_MOESM4_ESM.zip › MS_data_GFP-GANATA5-Glc_pHT059.pdf]

M.Tomek 12433: 10.993.1, 1:5, 3ul inj C4

20190626mt\_12433\_109931 564 (9.646)

1: TOF MS ES+  
1.53e7

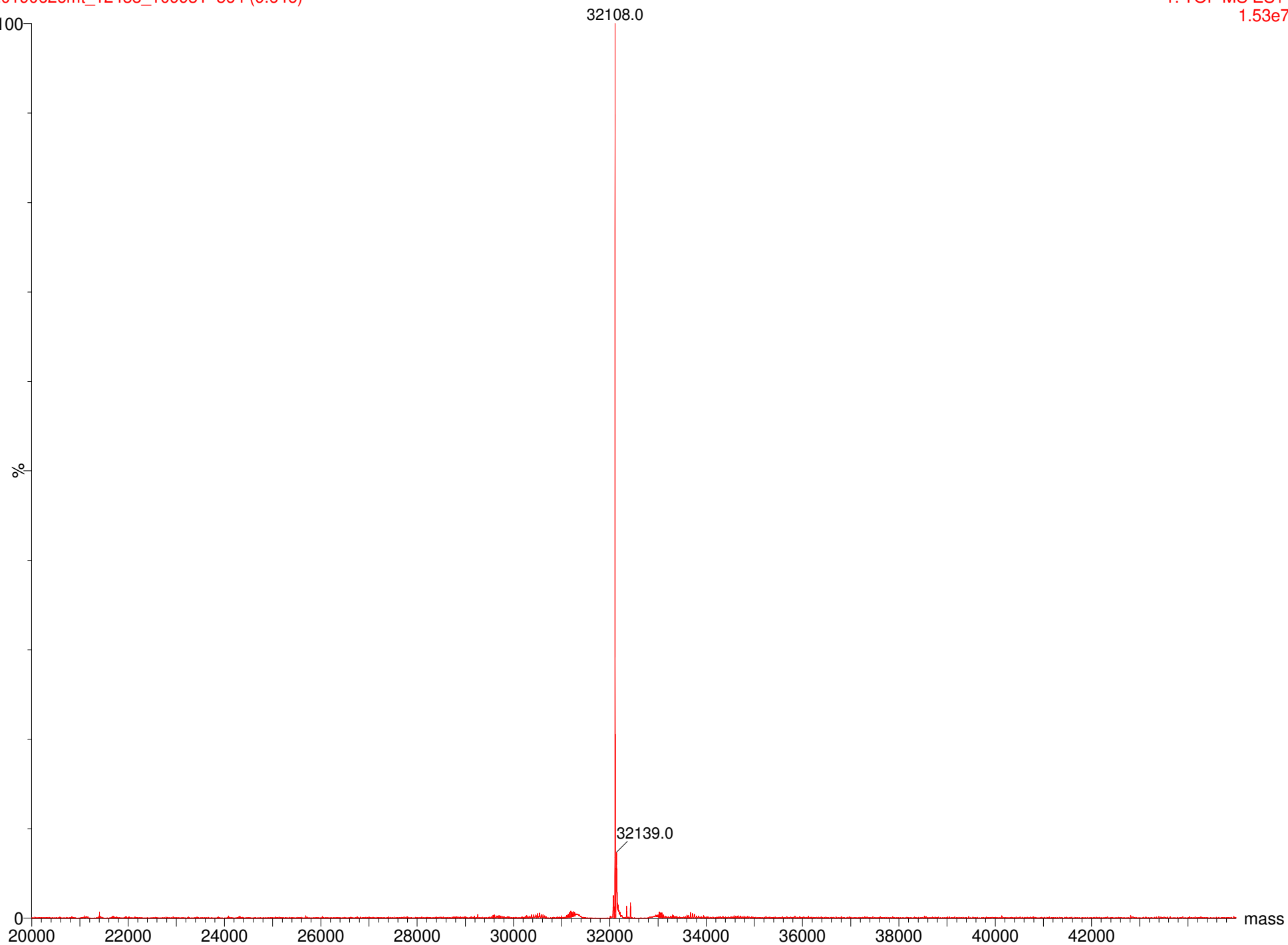

M. Tomek 12464: dil. 1:10: 2 ul C4 ZT: 010.993.2

20190903MT\_12464\_0109932 126 (2.147)

TOF MS ES+  
7.28e7

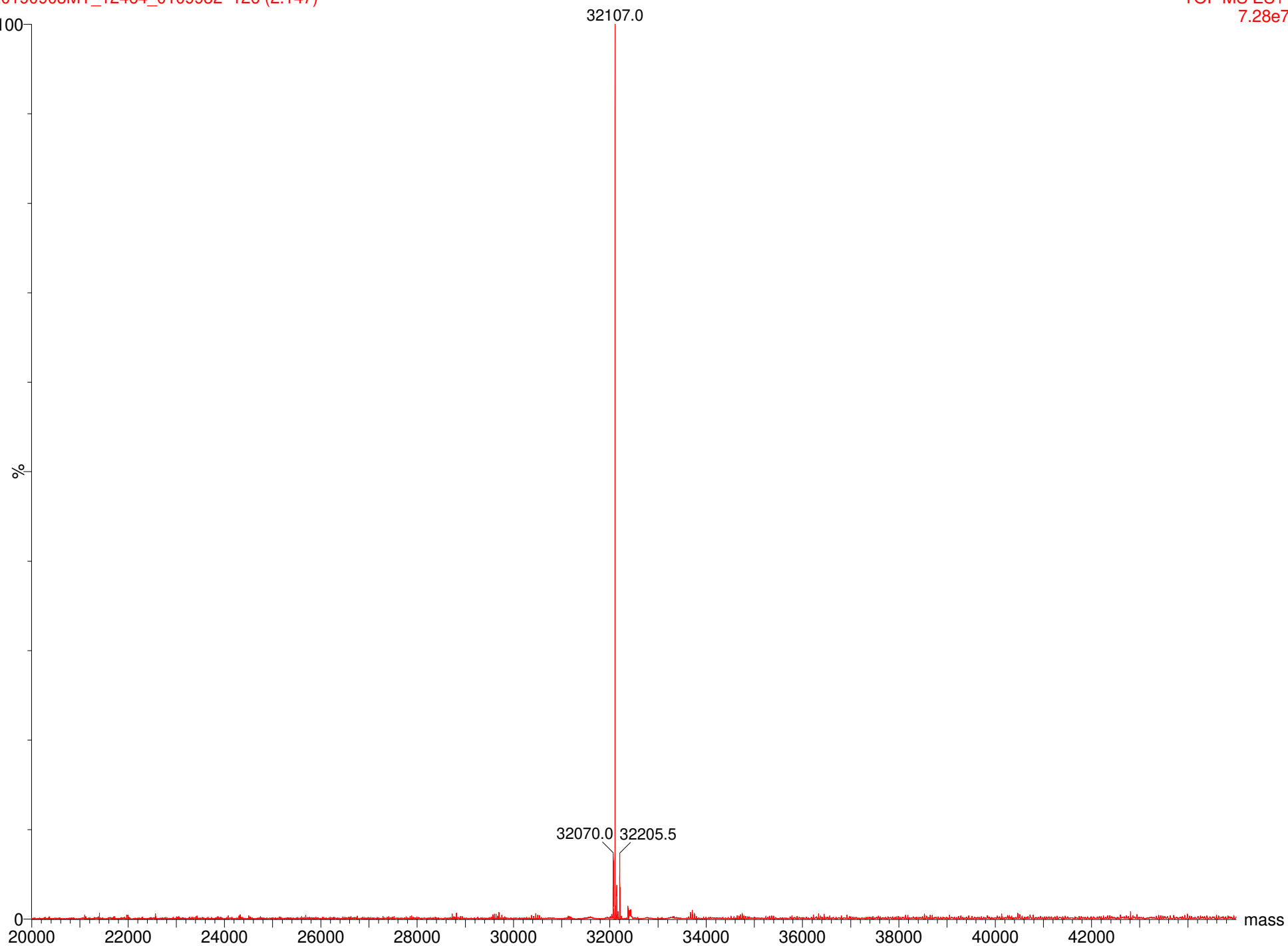

M. Tomek 12464: dil. 1:10: 2 ul C4 ZT: 010.993.3

20190903MT\_12464\_0109933 87 (1.487)

TOF MS ES+  
1.09e8

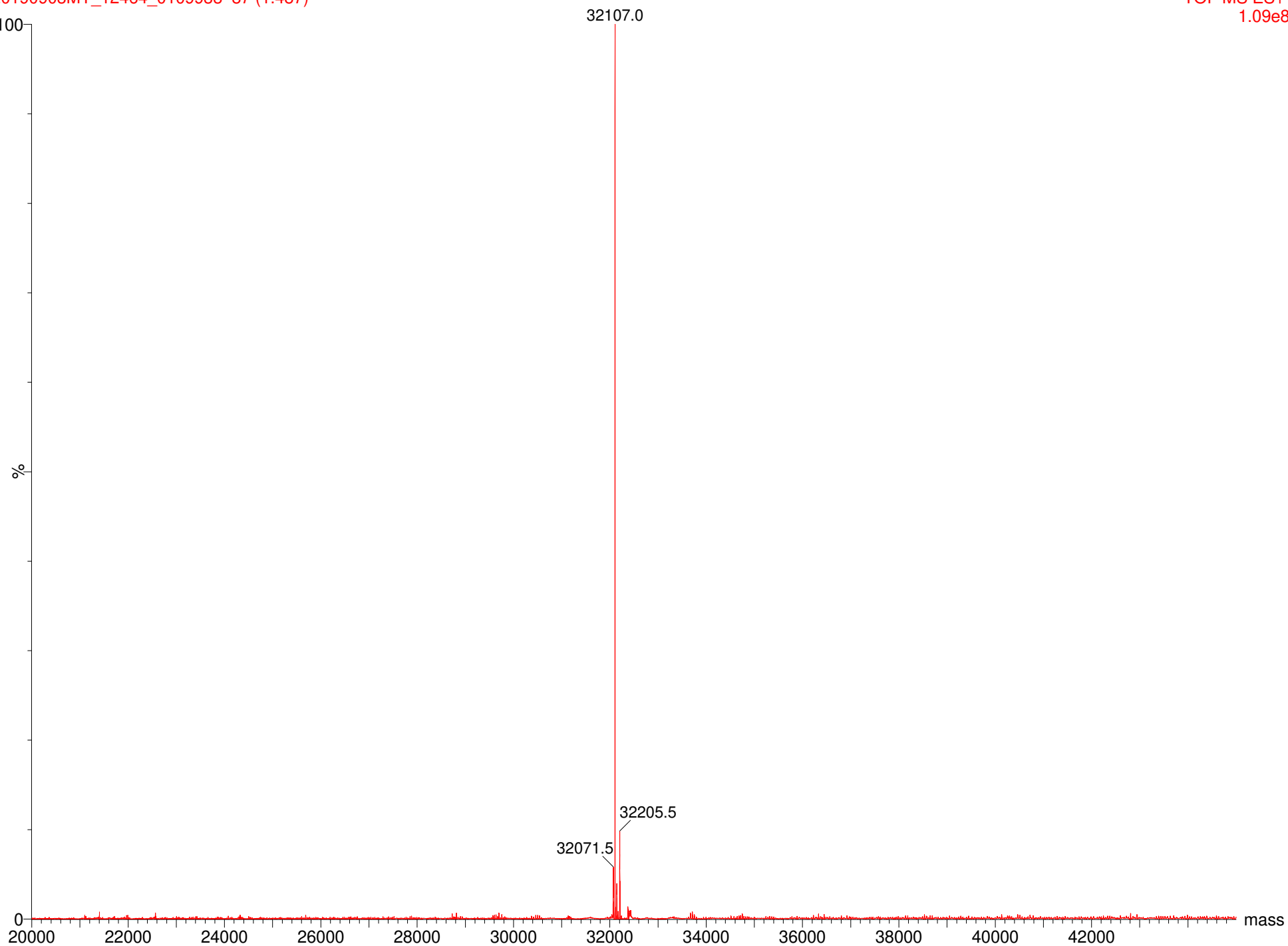

Supplement: Supplementary file 4 — Source Data [file 41467_2019_13283_MOESM4_ESM.zip › MS_data_GFP-GANATA5-Glc_pMA993.pdf]

M.Tomek 12433: 10.59.1, 1:5, 3ul inj C4

20190626mt\_12433\_10591 562 (9.611)

1: TOF MS ES+  
7.32e6

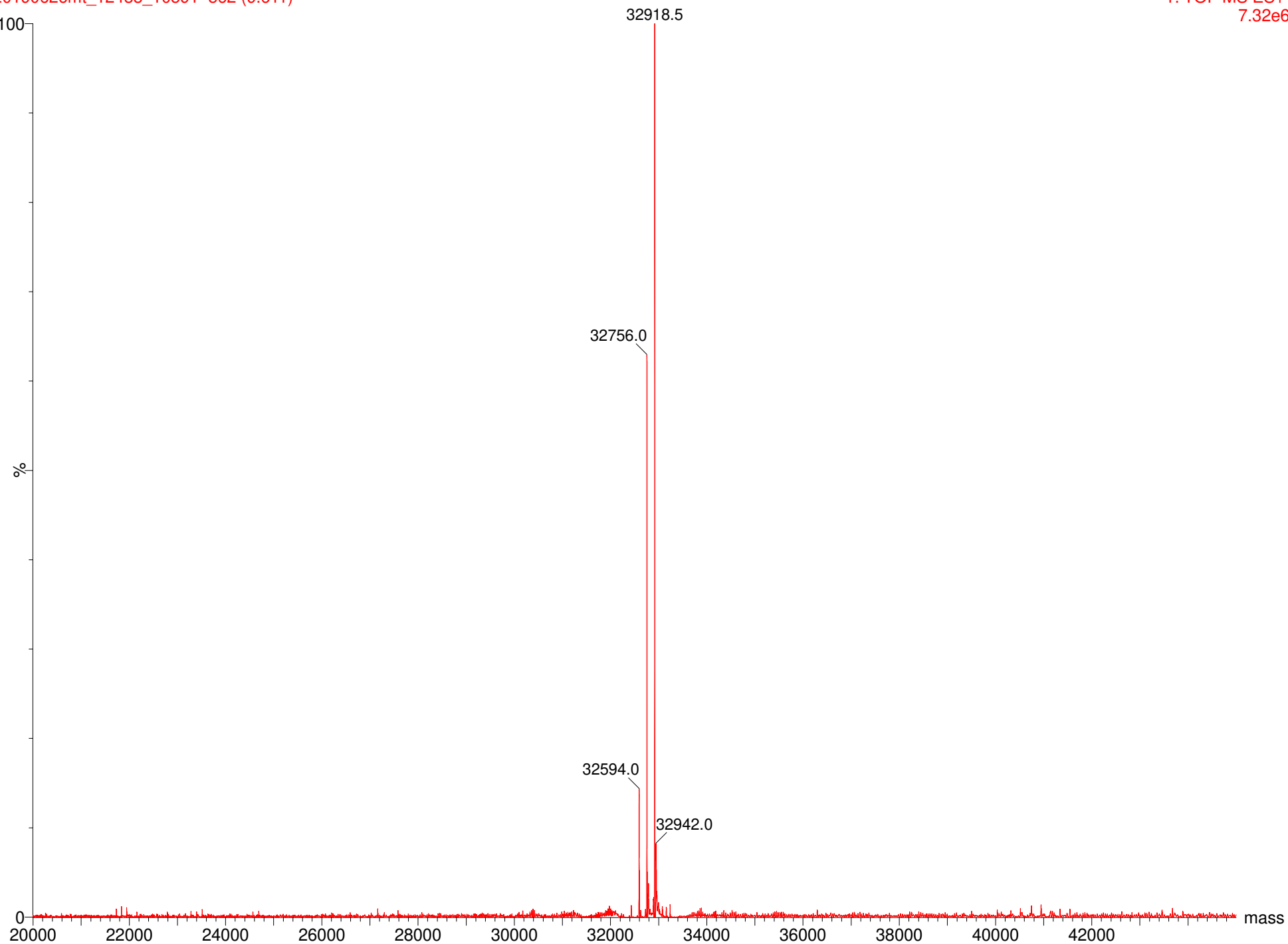

M.Tomek 12436: 010.60, 1:5, 3ul inj C4

20190626mt\_12436\_010\_60 563 (9.629)

1: TOF MS ES+  
5.77e6

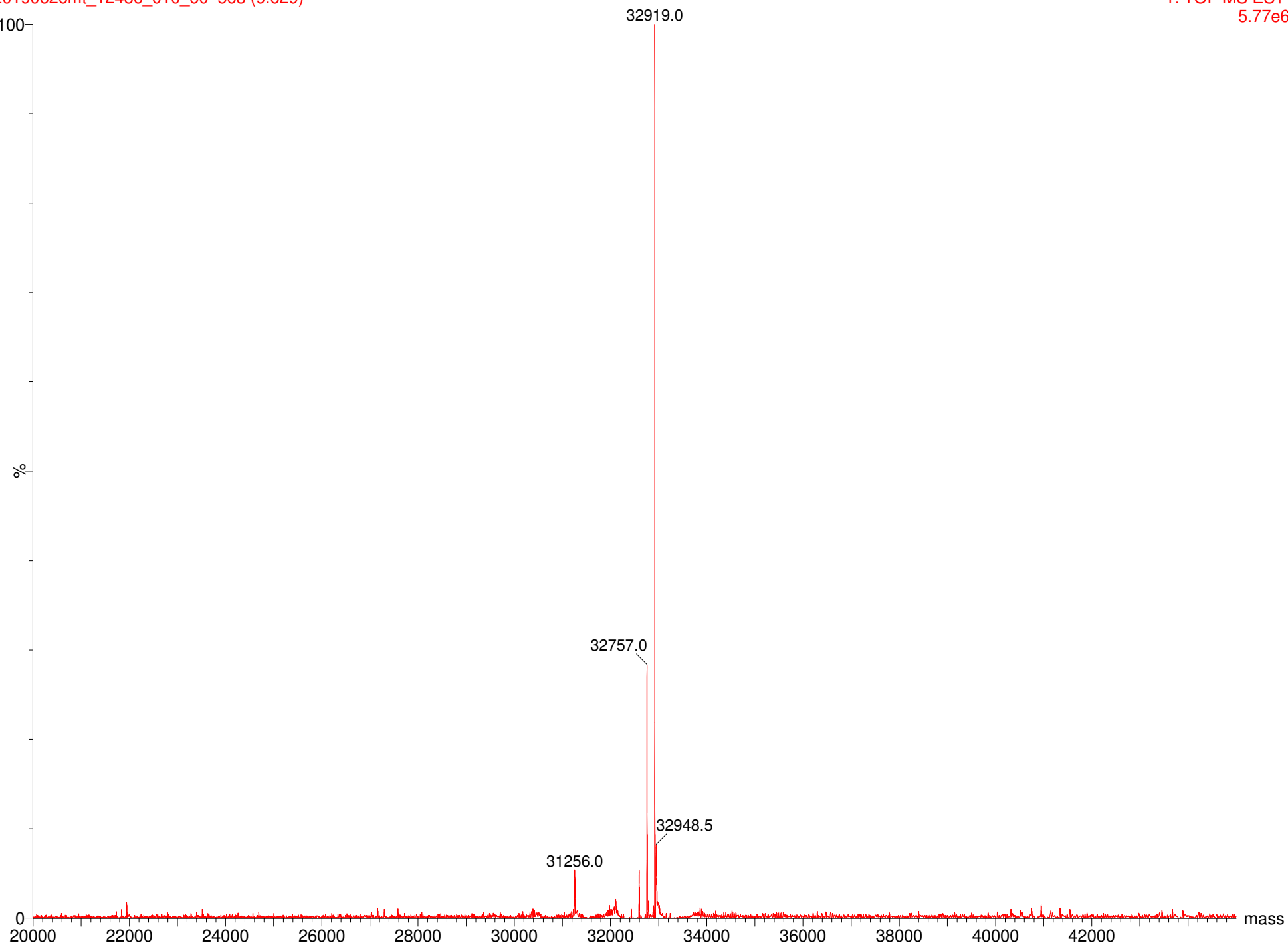

Supplement: Supplementary file 4 — Source Data [file 41467_2019_13283_MOESM4_ESM.zip › MS_data_GFP-GANATA5-Lac.pdf]
